# Increasing the broad-leaved tree fraction in European forests mitigates hot temperature extremes

**DOI:** 10.1038/s41598-020-71055-1

**Published:** 2020-08-25

**Authors:** Jonas Schwaab, Edouard L. Davin, Peter Bebi, Anke Duguay-Tetzlaff, Lars T. Waser, Matthias Haeni, Ronny Meier

**Affiliations:** 1 ETH Zurich, Institute for Atmospheric and Climate Science, Zürich, Switzerland; 2 WSL Institute for Snow and Avalanche Research SLF, Davos Dorf, Switzerland; 3 Federal Office of Meteorology and Climatology MeteoSwiss, Zürich-Airport, Switzerland; 4 Land Change Science, Swiss Federal Institute for Forest, Snow and Landscape Research WSL, Birmensdorf, Switzerland; 5 Forest Dynamics, Swiss Federal Institute for Forest, Snow and Landscape Research WSL, Birmensdorf, Switzerland

**Keywords:** Climate sciences, Ecology, Environmental sciences

## Abstract

Forests influence climate through a myriad of chemical, physical and biological processes and are an essential lever in the efforts to counter climate change. The majority of studies investigating potential climate benefits from forests have focused on forest area changes, while changes to forest management, in particular those affecting species composition, have received much less attention. Using a statistical model based on remote sensing observations over Europe, we show that broad-leaved tree species locally reduce land surface temperatures in summer compared to needle-leaved species. The summer mean cooling effect related to an increase in broad-leaved tree fraction of 80% is relatively modest (~ 0.3–0.75 K), but is amplified during exceptionally warm periods. The reduction of daily maximum temperatures during the hottest days reaches up to 1.8 K in the Atlantic region and up to 1.5 K in Continental and Mediterranean regions. Hot temperature extremes adversely affect humans and ecosystems and are expected to become more frequent in a future climate. Thus, forest management strategies aiming to increase the fraction of broad-leaved species could help to reduce some of the adverse local impacts caused by hot temperature extremes. However, the overall benefits and trade-offs related to an increase in the broad-leaved tree fraction in European forests needs to be further investigated and assessed carefully when adapting forest management strategies.

## Introduction

Forests are expected to play an essential role in climate change mitigation as they can generally sequester more carbon than non-forested ecosystems^1–3^. In addition, forests affect water and energy fluxes at the earth surface through biogeophysical processes including changes in evapotranspiration, albedo, and surface roughness^4,5^. Various observation-based studies have shown that forests, through these biogeophysical processes, either reduce or increase local temperatures depending on location and time of observation^6–9^. In contrast to a comparison of forested and non-forested ecosystems, the potential impacts on temperatures of forest management or more generally changes in forest characteristics are less well documented^10–12^.

Facilitating an increase of the broad-leaved tree fraction (BTF) in forests is a promising management strategy to enhance the provision of ecosystem services and to adapt to climate change^13–16^. For example, increasing the BTF can lead to reduced risk of fires, wind throw and bark beetle outbreaks^15,17^. However, the potential benefits of broad-leaved trees through their biogeophysical influence on temperature, in particular on extreme temperatures, have not yet been investigated beyond the site-level scale^18^ even though changes on extreme temperatures are highly relevant in terms of impacts on humans and ecosystems^19,20^.

To investigate how an increase in the BTF in Europe would influence local land surface temperature (LST) we linked observed patterns of LST with patterns of the BTF. In contrast to previous studies, we use remote sensing LST observations with a high sub-daily temporal resolution from the SEVIRI (Spinning Enhanced Visible and InfraRed Imager) instrument, which are a prerequisite for better understanding how forest cover changes influence daily maximum temperatures of exceptionally hot days. We also employ LST data based on MODIS (Moderate Resolution Imaging Spectroradiometer) in order to investigate the dependency of our results regarding the choice of LST data. We use Generalized Additive Models^21^ as a novel approach for analyzing how land cover influences temperature. GAMs allow to explicitly model the non-linear relationship between temperature, land cover, latitude, longitude, elevation and additional topographic variables. Since GAMs have an additive structure, analyzing the effect of the BTF on temperature can be roughly understood as expressing the observed LST as a function of topography (e.g. elevation) as well as latitude and longitude (being a proxy for the general weather situation) and correlating the unexplained temperature signal with land-cover data, in particular the BTF (Methods and Supplementary Information).

## Results

**Figure 1:**
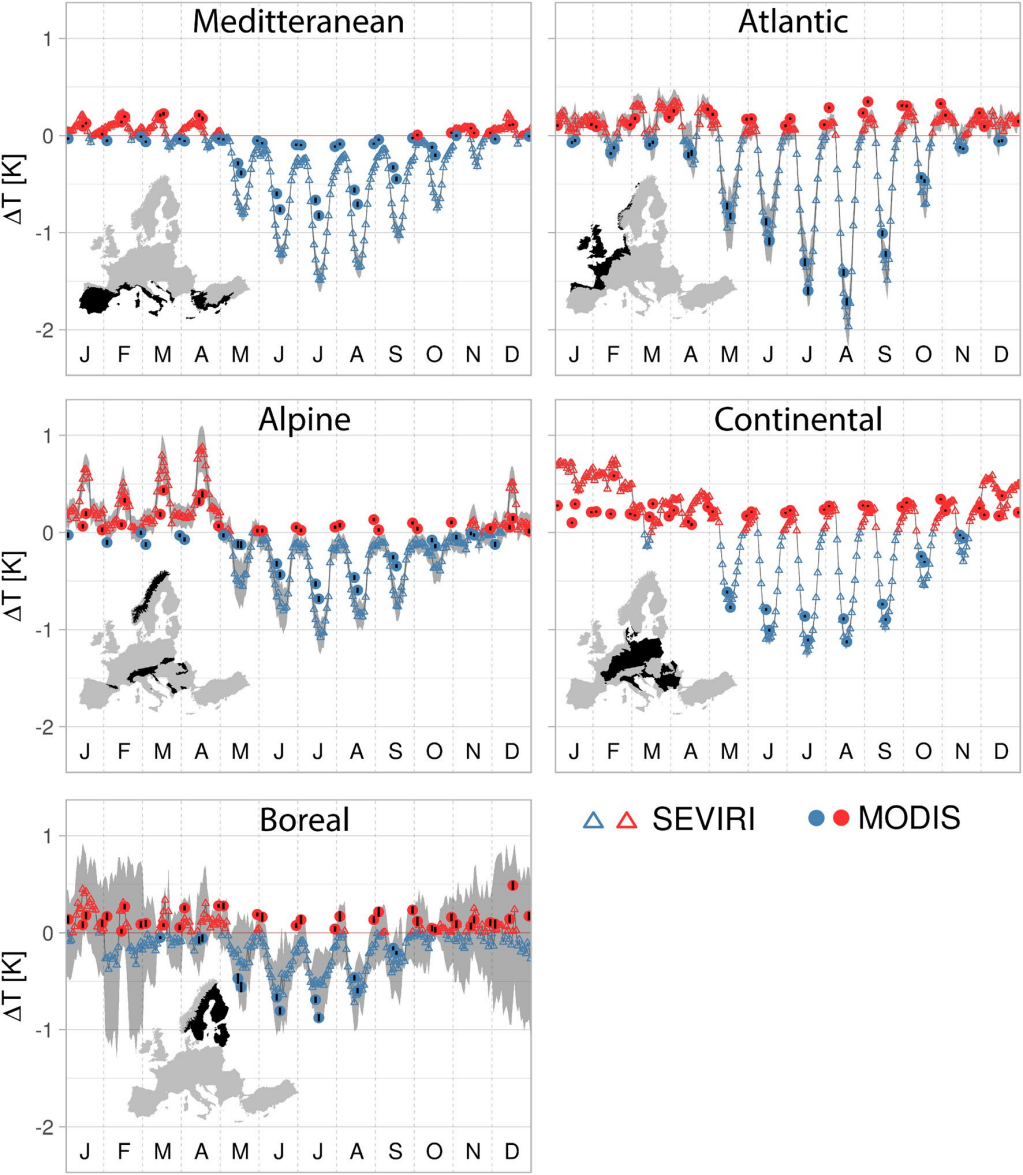
Pronounced mean diurnal and seasonal cycles of temperature changes caused by an increase in BTF reveal a cooling effect in summer. For every month denoted as J (January), … , D (December) on the x-axis the mean diurnal cycle of the LST change induced by an 80% increase of the broad-leaved tree fraction is shown. For SEVIRI hourly mean values are available (i.e. 24 values for each month). For MODIS, observations are available at four different time steps per day which are approximately: 01:30, 10:30, 13:30 and 22:30. Confidence intervals for SEVIRI are shown in grey. Confidence intervals for the MODIS observations are denoted as black lines through the colored dots.

We found that an increase of the BTF from 10 to 90% results in pronounced diurnal and seasonal cycles of LST changes (Fig. 1). During summer days, an increase in BTF induces a cooling in all five biogeographical regions. With up to 2 K in August, the cooling is largest in the Atlantic region. In the four other regions the cooling lies roughly between 0.5 and 1.5 K. At nighttime, there is a slight warming effect throughout the whole year except for the Mediterranean. In winter either cooling or warming occur for different regions during the day.

The signals derived from MODIS and SEVIRI strongly agree for most regions despite the differing resolutions and methods used to produce the two LST products. An exception is the Mediterranean, where the summer daytime cooling effect is much more pronounced for SEVIRI (up to 1.5 K) than for MODIS (up to 0.85 K). An assessment of the mean temperature response shows that seasonal and diurnal cycles largely average out and that the annual mean temperature response in several regions is close to zero (Fig. S1). In addition, we find that considering the two observations per day provided by MODIS (Aqua satellite; approximate acquisition times 13:30 and 01:30) can roughly approximate the annual mean effect of an increasing BTF on LST. Daily maximum temperatures in most regions occurred earlier over broad-leaved than over needle-leaved forests (Fig. S2) indicating that an increase in broad-leaved tree fraction also alters the diurnal temperature cycle.

**Figure 2:**
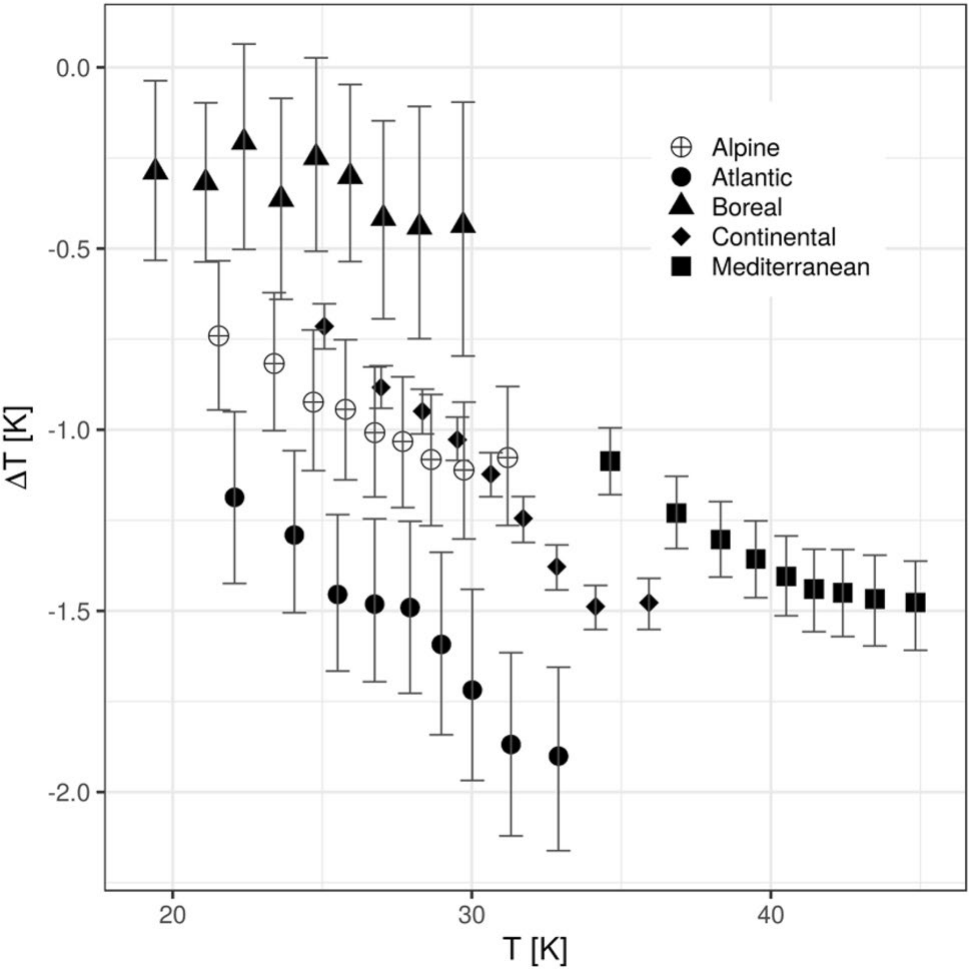
During exceptionally high temperatures the cooling provided through an increase in BTF is amplified. The LST change (based on SEVIRI LST) related to an increase of 80% in BTF is plotted against the temperatures of the different quantiles (i.e. temperature change for moderate background temperature (0.1 quantile) and very high background temperature (0.9 quantile)). The error bars show the 95% confidence interval.

To assess the cooling effect in summer for high temperature extremes we separated the observed daily maximum temperatures into quantiles. Within each biogeographical region the cooling effect in summer is larger for higher daily maximum temperatures represented by higher quantiles (Fig. 2). Even though the cooling potential within each region is clearly related to the background temperature, the cooling potential between different regions is not. For example, the highest temperatures are observed in the Mediterranean (Fig. 2). However, the cooling potentially provided by an increase in BTF in the Mediterranean is lower than the one in the Atlantic region and similar to the one in the Continental region for high quantiles.

## Discussion

### Regional differences and biophysical processes

The lower temperature prevailing in broad-leaved compared to needle-leaved forests during summer can be linked to the larger fraction of shortwave radiation reflected by broad-leaved trees (i.e. a higher albedo^9,22,23^) and the generally larger latent heat flux over broad-leaved forests due to higher stomatal conductance^9,24,25^. Regional differences may originate from the interplay between these radiative and non-radiative effects. For instance, the cooling signal in boreal forests is moderate in comparison to other regions. This may be related to lower incoming shortwave radiation reducing the impact on temperature of the higher albedo of broad-leaved forests. In contrast, in the Mediterranean the amount of incoming shortwave radiation is high, even more so under clear-sky conditions prevailing during the hottest summer days, which results in a potentially larger sensitivity to albedo differences ^26^. At the same time, broad-leaved tree species in the Mediterranean have evolved to cope with dry summer conditions by adopting structural and physiological attributes (e.g. smaller leaves) that lead to lower rates of evapotranspiration^27^ and hence potentially smaller evapotranspiration differences between broad- and needle-leaved species^28^. In contrast, differences in evapotranspiration in the Atlantic region, which is characterized by an oceanic climate, may be more pronounced between broad-leaved and needle-leaved species. As soil moisture is less of a limiting factor^29,30^, differences in evapotranspiration due to physiological traits of broad- and needle-leaved species may become more prominent considering as well that broad-leaved species are naturally predominant and well adapted to environmental conditions in this region^31,32^. The lower cooling signal in the Continental region compared to the Atlantic region might also be related to lower differences in evapotranspiration due to less water availability in summer. However, to better understand radiative and non-radiative processes for different regions further detailed analysis of species specific traits will be crucial.

We observe that the summertime cooling of an increased BTF in the Mediterranean region hardly increases between the 0.6 and 0.9 temperature quantiles. A possible explanation could be that soil moisture may be very low for high temperature quantiles. Because of this, the differences in evapotranspiration between broad-leaved and needle-leaved forests, which may partly result from a higher stomatal conductance of broad-leaved trees^33^, could be attenuated due to soil moisture limitation. Hence, an increased warming and drying in the Mediterranean^34^ may not further increase or even decrease the cooling potential of more broad-leaved trees. This indicates that forest management efforts like thinning, the promotion of different types of mixed forests and changing species composition could be more relevant in such regions^35,36^. In contrast to the Mediterranean, the projected increase in temperature in the Boreal region^34^ may enhance the cooling potential of broad-leaved trees. However, in some boreal regions (e.g. southern Sweden) low soil moisture in summer is already limiting forest growth and evapotranspiration^37^. This may be one of the reasons for the low temperature dependency of the observed cooling potential of broad-leaved forests in boreal regions (Fig. 2). It has to be kept in mind for all regions that a higher evapotranspiration of broad-leaved trees^38^ could potentially lead to an accelerated soil moisture depletion, which has been shown to be relevant for high versus low density forest stands and when comparing forests to grassland^39–41^. On the other hand, broad-leaved species have been reported to shift water uptake to deeper soil layers than certain needle-leaved species (e.g. spruce), which may allow broad-leaved trees to sustain higher evapotranspiration even during drought conditions^42,43^. Accordingly, evidence provided for central Europe shows that broad-leaved stands (beech vs. spruce) were able to maintain a lower ratio between sensible and latent heat fluxes even during drought stress^44^, confirming that increasing the broad-leaved tree fraction in forests could indeed reduce the severity of heatwaves at a local level.

### Diurnal cycle of LST differences

The temperature differences between broad-leaved and needle-leaved forests in summer exhibit a pronounced diurnal cycle. During nighttime, temperature differences between the two forest types are often close to zero whereas they are highest during daytime. During daytime, energy fluxes are driven by incoming solar radiation^45^. The lower albedo and higher evapotranspiration of broad-leaved forests likely lead to lower sensible heat fluxes and temperatures. During nighttime the lack of incoming shortwave radiation implies that shortwave radiation and latent heat fluxes play a minor role^46^ and that differences in the impact of the two types of forests on the energy balance are small. Daily maximum temperatures in most regions occurred earlier over broad-leaved than over needle-leaved forests (Fig. S2). Accordingly, temperature differences between broad-leaved and needle-leaved trees can reach their maximum after the absolute maximum temperatures over forests are observed (Figs.S3, 1). A possible explanation for the differences in the diurnal cycles is that coniferous tree species close stomata earlier than many deciduous broad-leaved tree species^47–49^. Thus, there may be a stronger decrease in latent heat fluxes over needle-leaved trees leading to higher cooling provided by broad-leaved trees in the afternoon.

### Comparison with in-situ observation

The results based on remote sensing data are compared to in-situ observations from 10 different paired sites (forest versus open land, where forest is either characterized by needle-leaved or broad-leaved trees) located in Switzerland, which is part of the Alpine region^50^. In situ-observations corroborate the findings that the cooling potential provided by broad-leaved trees increases from lower to higher maximum daily temperature in summer (Fig. S4). The diurnal and seasonal cycles of differences in temperature between broad-leaved and needle-leaved sites in the Alpine region agree well with the in-situ observations in Switzerland (Fig. S3). However, it is important to note here some of the limitations of such comparison. By subtracting the temperatures at forest sites from the ones at very similar open-land sites for each paired observation, we are able to partly remove the impact of topography and other environmental characteristics on the temperature signal. However, there may still be some systematic differences between broad-leaved and needle-leaved paired sites. Such differences could explain why the analysis of the station data suggests lower nighttime temperature for broad-leaved trees, whereas the analysis of remote sensing data shows a very week or even slightly positive nighttime signal. It is also important to note, that the different broad-leaved and needle-leaved species encountered at the paired sites show very distinct temperature signals and dividing them into just two categories masks these differences (cf. Supplementary Information^48^). In addition, the in-situ observations measure 2 m air temperature within the forest canopy and not land surface temperature as observed by satellites, which means that the temperature signals have to be compared carefully^51^.

### Co-benefits and trade-offs related to an increased BTF

Increasing the broad-leaved tree fraction may be related to trade-offs that need to be taken into account when assessing whether this is a suitable strategy for a specific region. A recent study in the European Alps has shown that an increased evapotranspiration during heatwaves over vegetated areas could reduce run-off and increase water-scarcity, in particular at high elevations^52^. Considering that broad-leaved trees tend to have higher evapotranspiration rates than needle-leaf trees, increasing the number of broad-leaved trees may contribute to water scarcity in similar situations. However, needle-leaved trees tend to intercept more precipitation^53–55^, which is partly evaporated before reaching the soil. Thus, discharge towards surface and groundwater is generally higher for broad-leaved species and soils of broad-leaved forests are wetter^10^, which would partly counteract negative impacts on water availability and needs to be further investigated. Adaptive forest management will also require site-specific information about the suitability of various tree species. For example, it is difficult to assess in general if either broad-leaved or needle-leaved tree species will cope more successfully with an increasing frequency and severity of droughts^25^. In general, increasing the broad-leaved tree fraction may be most beneficial in regions, where natural broadleaved forests have been replaced by non-native coniferous plantations^17,56,57^, but not in those regions where conifers tend to be become dominant over broad-leaved trees under harsh environmental conditions as, e.g., in subalpine forests^58^.

Potential benefits of an increased BTF are a decreased risk of fires, wind throw and bark beetle outbreaks^15,17,59^. An increase in the BTF may be particularly beneficial if it leads to higher biodiversity^16,60^. Thus, rather than a complete conversion from needle-leaved to broad-leaved-based forests, a more moderate increase in BTF preserving species diversity may perform better across various sustainability indicators^14^. Such a moderate increase would still have a substantial impact on LST, since it decreases approximately linear with the increase of the BTF (Fig. S5). The economic benefits of mixed forests can also be higher than the ones of mono-species forests taking into account that purely coniferous forests are often more susceptible to large-scale natural disturbances^14^. These disturbances can strongly affect ecosystem services including the release of greenhouse gases, reduced protection against natural hazards and a higher risk of decreased timber yields^61^. Despite potential climatic, ecological, and economic benefits, forest managers could be reluctant to increase the number of broad-leaved tree species in the absence of monetary incentives, which might be sometimes necessary to trigger a change in the market and the production system^15^.

## Conclusions

Overall, our results suggest that European forests with a high BTF exhibit lower LSTs during hot extremes in summer than forests having larger fractions of needle-leaved trees. Thus, increasing the BTF could help to locally reduce some of the adverse impacts of hot temperature extremes^62^. A reduction of daily maximum temperatures during hot days of up to 1.8 K, as indicated by our results, can have important implications since the negative impacts of hot temperatures on humans and ecosystems often increase nonlinearly with increasing temperature^63–65^.

Since our current understanding of all the relevant synergies and trade-offs related to a change in forest composition is still incomplete, further research integrating results from various fields is needed. Two challenges regarding the biophysical climate impacts of forests will be to assess species-specific impacts on temperatures and how shifting environmental conditions will affect the summertime cooling potential of different broad-leaved species.

## Methods

### Land surface temperature (LST)

Remote sensing LST products derived from different satellites and two different instruments, MODIS (Moderate Resolution Imaging Spectroradiometer) and SEVIRI (Spinning Enhanced Visible and InfraRed Imager) were used and are referred to as MODIS-LST and SEVIRI-LST. Both, MODIS-LST and SEVIRI-LST were downloaded for the European domain (Supplement S 11) between 01/01/2009 and 31/12/2014. Since the two LST datasets are based on raw data from different instruments, mounted on different satellites, we were able to assess uncertainties potentially related to the different LST products.

The MODIS instrument onboard a polar orbiting satellite permits the production of high spatial resolution LST (~ 1 km) at a temporal resolution of 4 observations per day. Raw data from the MODIS sensor is the basis for several LST products. In this study we use version 6 of the two products MOD11A2 and MYD11A2 which provides an 8-day average LST on 1 km spatial resolution^66^. MOD11A2 and MYD11A2 are available at two different (solar-) times during the day (MOD11A2 ~ 10:30 and ~ 22:30, MYD11A2 ~ 13:30 and ~ 01:30). Both products rely on a sinusoidal projection and were transformed into the European coordinate system ETSR89. The retrieval of land surface temperature from satellite instruments requires a priori knowledge of the atmospheric state and the surface emissivity. Hence, satellite-based land surface retrievals have uncertainties. MODIS LST products have been validated^66^ with a series of field campaigns and in radiance-based validation studies. Accuracy of MODIS LST is generally better than 2 K. Larger bias can occur due to heavy dust loads, aerosols and uncertainties in classification-based surface emissivity. Pixels with an emissivity error estimate larger than 0.04 and/or an LST error estimate larger than 3 K were excluded from the analysis.

In contrast, LSA SAF SEVIRI-LST is based on a geostationary satellite allowing LST to be produced at a high temporal resolution of 15 min intervals, but on a lower spatial resolution of about 3-5 km^67^. Reported uncertainties for the LSA SAF LST dataset are in the range of 1 to 2 K^68^, except for very moist atmospheres and satellite viewing angles exceeding 50°^69^. Surface emissivity is estimated based on land cover classes and the fraction of vegetation cover^67^. First, pixels of errors larger than 3 K and pixels that were corrupted according to the quality control data were removed. Thereafter the data were georeferenced and transformed to the World Geodetic System 1984 (WSG84). Time reference of SEVIRI-LST is UTC. To allow for a comparison with the MODIS data, which is provided in solar time, we transformed the SEVIRI-LST data. For this purpose, we first calculated mean solar time at every location in Europe given a specific UTC time. The SEVIRI-LST solar time grids were then split into longitudinal slices, each spanning a 15 min interval. All slices matching a specific solar time (i.e. slices from different grids) were recombined into one grid so that solar time was reasonably well approximated. As a last step the data was transformed to European coordinates (ETSR89) creating a regular grid with a resolution of 3.8 km.

### Forest cover data

Information on the broad-leaved tree fraction was based on data provided by the Copernicus Land Monitoring Service. The data are called High Resolution Layers (HRLs) and contain information on forest type. We used this dataset available for 2012 containing binary information on whether a grid cell (20 m × 20 m) is mainly covered by broad-leaved trees or mainly covered by coniferous trees (which we refer to as needle-leaved trees). Raw data used for the generation of the HRL layers include multispectral time-series of Sentinel-2A, Landsat 8, SPOT-5 and ResourceSat-2 satellite data. Further details are provided in the product specification document by Copernicus^70^. The data was resampled to match the resolution of the transformed MODIS (1 km) and SEVIRI LST (3.8 km), before being used in the statistical analysis (Fig. S6).

### Additional environmental attributes

Data on environmental attributes were included as predictor variables to account for possible confounding factors in the Generalized Additive Models (Tables S1, S2 and Fig. S6). The digital elevation model EU-DEM v1.0 ^71^ was used to include elevation as a predictor and to calculate Slope and Aspect (using the functions “Slope” and “Aspect” as part of the Spatial Analyst tool provided by ESRI, ArcGIS Desktop 10.5.1). The data on aspect was reclassified into two categories: South facing slopes (90°–270°) and north facing slopes (270°–360° and 0°–90°). Based on this categorization we calculated the fraction of north facing slopes for each grid cell. As an indicator for further topographic properties of each grid cell we calculated a Terrain Ruggedness Index (TRI) and a Topographic Position Index (TPI) using the tool gdaldem available within the Geospatial Data Abstraction software Library 2019 (GDAL). The terrain ruggedness index is defined as the mean difference in elevation between a central grid cell and all its surrounding cells. The Topographic Position Index is calculated as the elevation difference between a central grid cell and the mean elevation of its surrounding cells. In addition to topographic attributes we included information on land cover based on CORINE Land Cover (CLC) data^72^. The CLC data is provided for 3 different hierarchical levels of which the first one includes 5 land cover categories, the second one 15 and the third one 44 (Table S2). We included information for 15 different categories, which allowed us to distinguish in detail between different land covers without including unnecessary predictors in the statistical model.

### Calibration and setup of generalized additive models (GAMs)

The potential change in temperature that could result from an increased broad-leaved tree fraction was assessed by fitting Generalized Additive Models (GAMs) using the previously described predictors and LST data (Table S1). We calibrated the models for 5 biogeographical regions in Europe (Mediterranean, Atlantic, Alpine, Continental and Boreal), assuming the effect of an increasing BTF would be constant within each region. In an additional experiment we allowed the BTF effect to vary with latitude and longitude to get additional insight into potential sub-regional variations of the temperature change induced by an increase of the BTF.

To separate the potential change in temperature related to an increase in BTF during hot extremes from the change during less extreme temperatures, we aggregated the LST records in the following way: We used the SEVIRI LST data in summer (JJA) between 2009 and 2014 and calculated the maximum temperature for each day and each pixel. The daily maximum temperatures in each pixel were then reordered into 9 quantiles (0.1, 0.2, … , 0.9) separating the observations during hot days (high quantiles) from the observations during less hot days (lower quantiles). This means that we used 9 response variables (namely for each quantile, Fig. S7) when fitting the General Additive Models, which can be described as:

1 $${\mu }_{i}= {f}_{1}{(BTF}_{i}) + {f}_{2}\left({X}_{i},{Y}_{i}\right) + ... + {f}_{7}\left({TPI}_{i}\right)+ {f}_{8}\left({V}_{1,i}\right)+ ...+{f}_{22}\left({V}_{14,i}\right)$$

where ${\mu }_{i}$ = $E({y}_{i})$ and ${y}_{i}$ is from a scaled t-distribution, which was chosen because of the heavy tails of the response variable (i.e. LST). A smooth function of each predictor (Table S1) was included as an additive term. The LULC data consisting of 15 land-cover categories was transformed into the variables ${V}_{1}$ to ${V}_{14}$ before including the LULC information into the model (Supplement S 22). The geographic coordinates ($X, Y$) were modelled as two-dimensional tensor product smooths. All other functions $f$ were represented using thin plate regression splines. The statistical analysis were performed in the R computing environment^73^ using the package mgcv to fit all GAM models^21^. In particular, we used the function bam, which allows for parallel computing and has the advantage of a low memory footprint^74^. The method used for the smoothing parameter estimation was fREML (fast restricted maximum likelihood). Since we rely on a model for a nonexponential family (scaled t distribution) an updated version of the mgcv package was used^75^.

To further analyze the temporal pattern of temperature changes induced by an increase in BTF, we relied on the previously described setup, but introduced further response variables to get an understanding of the diurnal and seasonal cycle. To this account we calculated multi-annual mean land surface temperatures (from 2009 to 2014) for every month and the diurnal cycle of SEVIRI-LST and the four daily observations available for MODIS-LST. The majority of studies analyzing the impact of land-use and land management changes on land surface temperature rely on MODIS-LST^6,9^. Since we include data on higher temporal resolution we were able to test whether the lower temporal resolution of MODIS would matter when calculating the mean temperature response of land-cover changes. This was done by calculating the mean effect relying on a limited number of SEVIRI observations (10:00, 13:00, 22:00, 01:00) that correspond roughly to the approximate retrieval times of MODIS (10:30, 13:30, 22:20, 01:30) and compare this effect with the one that was calculated including 24 observations per day.

All GAMs were diagnosed by checking residuals (including partial residuals), the basis dimension k, concurvity and by analyzing the goodness of fit (Supplement S3). In addition, we provide results for models in which we included less covariates and varied parameters like the basis dimension chosen to model the smooth terms (Figs. S8 and S9).

### Retrieving signals and uncertainties based on GAMs

The calibrated GAMs were used to predict the potential temperature change when increasing broad-leaved tree fraction by from 10 to 90% and was hence calculated as:

2 $$\Delta \mathrm{T}={T}_{90}-{T}_{10}$$

Variance and confidence intervals for $\Delta \mathrm{T}$ were estimated with the help of the prediction matrix ${X}_{p}$ and by simulating the posterior distribution of the parameters $\upbeta$ following Wood^21^. The matrix ${X}_{p}$ maps the model parameters $\widehat{\beta }$ to the predictions of the linear predictor ${\widehat{\eta }}_{p}$. After identifying ${X}_{p}$ in such a way that it satisfies ${X}_{p} \widehat{\beta }= {\widehat{\eta }}_{p}$, the linear response (i.e. LST) can be simulated for different parameter vectors drawn from the approximate posterior distribution of $\beta$. The difference between ${T}_{90}$ and ${T}_{10}$ (i.e. the temperature predicted for a BTF of 90% and 10%) was then estimated for 1,000 replicates of the parameter vector $\widehat{\beta }$. Based on the distribution of the LST differences we then calculated the confidence intervals for the 0.025 and 0.975 quantiles. Following this procedure allows inference about any sort of combination of the predictions which in our case is simply the difference between the two broad-leaved tree fractions. We chose the values 10% and 90% for the two scenarios, because pure needle-leaved or broad-leaved forests are relatively rare and hence predictions for values below 10% and particularly above 90% become more uncertain. In addition, we argue that pursuing a natural form of mixed-species forestry provides forest functions and services often to a higher level than monocultures^14^ and that disturbances as well as the variety of site characteristics will usually not lead to pure needle-leaved or broad-leaved stands^76^.

### In-situ observations

The results obtained based on remote sensing observations were compared against results from meteorological in-situ observations in Switzerland. Observations from 10 paired sites between 2009 and 2014 have been included. The data have been continuously recorded, calibrated and checked for quality^50^. Each paired site consists of one forest and one open land site, which are generally very close together (maximum distance 1.8 km). For every plot, information on forest species, elevation and orientation (i.e. aspect) are provided (Table S3). Our analysis of in-situ observations is closely related to previous studies relying on data from the same sites^48,77^. However, we specifically aim at comparing the results from the in-situ observations with results from the analysis of remote sensing data. This means that we focus on the same time steps as in the analysis of remote sensing data, we specifically focus on diurnal and seasonal cycles and how the temperature difference between broad-leaved and needle-leaved sites changes with background temperature in JJA.

We calculate the differences between temperatures measured at the forests sites and those measured at open-land sites. Since open-land sites and forest sites are almost equal in elevation and usually similar in terms of topography, calculating the differences between the two partly eliminates the effect of different topographies on temperature signals and allows to retrieve a land-cover signal. The differences between forest sites and open-land sites were averaged for all broad-leaved and needle-leaved sites and the resulting means were subtracted from each other. These temperature differences are summarized to display mean diurnal and seasonal cycles. In addition, the differences in JJA that were observed during the highest temperature of the day (according to the maximum temperature of each forest site) were aggregated for inter-quantile ranges (0.05–0.15, …, 0.85–0.95), to display the potential effect of a higher broad-leaved tree fraction during hot observations.

## Supplementary information

Supplementary information.
