# Supplementary material for: Increasing the broad-leaved tree fraction in European forests mitigates hot temperature extremes
Source: Sci Rep. 2020 Aug 25;10:14153. doi: 10.1038/s41598-020-71055-1 (PMC7447805; doi:10.1038/s41598-020-71055-1)
Supplement: Supplementary file 1 — Supplementary information. [file 41598_2020_71055_MOESM1_ESM.docx]

Supplementary Information

Figure S 1: Annual mean temperature signals caused by an increase in broad-leaved-tree fraction are relatively small and can be reasonably well approximated using two observations per day. Annual mean temperature response to an increase in BTF for different regions based on SEVIRI. Bars denote the annual mean temperature change, dots show the summer mean temperature change. Error bars denote the standard error of the mean. To test how well the mean can be approximated when using a limited number of observations per day, the mean temperature responses were calculated including either one observation (13:00), two observations (13:00 and 01:00), four observations (10:00, 13:00, 22:00 and 01:00) or 24 observations per day.


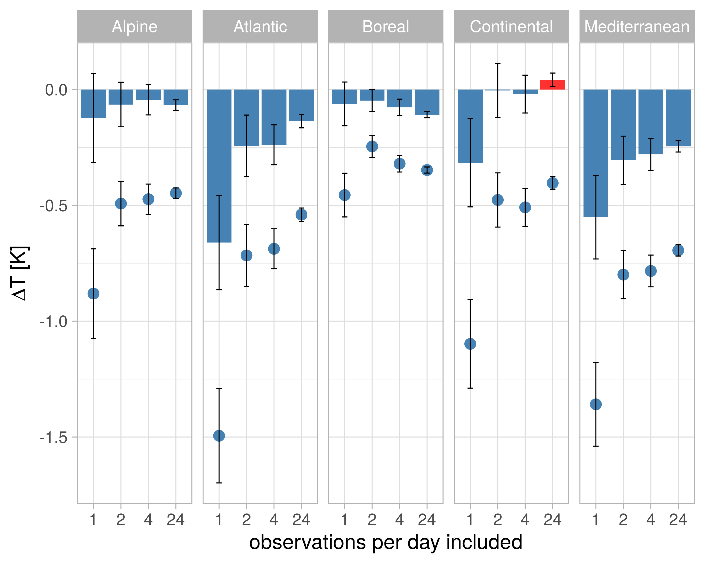


Figure S 2: Distribution of daily maximum temperatures for broad-leaved (B) and needle-leaved (N) forests in different regions and median values of each distribution. Forests were classified as broad-leaved if the BTF was higher than 75% and as needle-leaved if the BTF was lower than 25%. Pixels were regarded as forests if they were covered at least by 80% forest according to Corine Land Cover data.


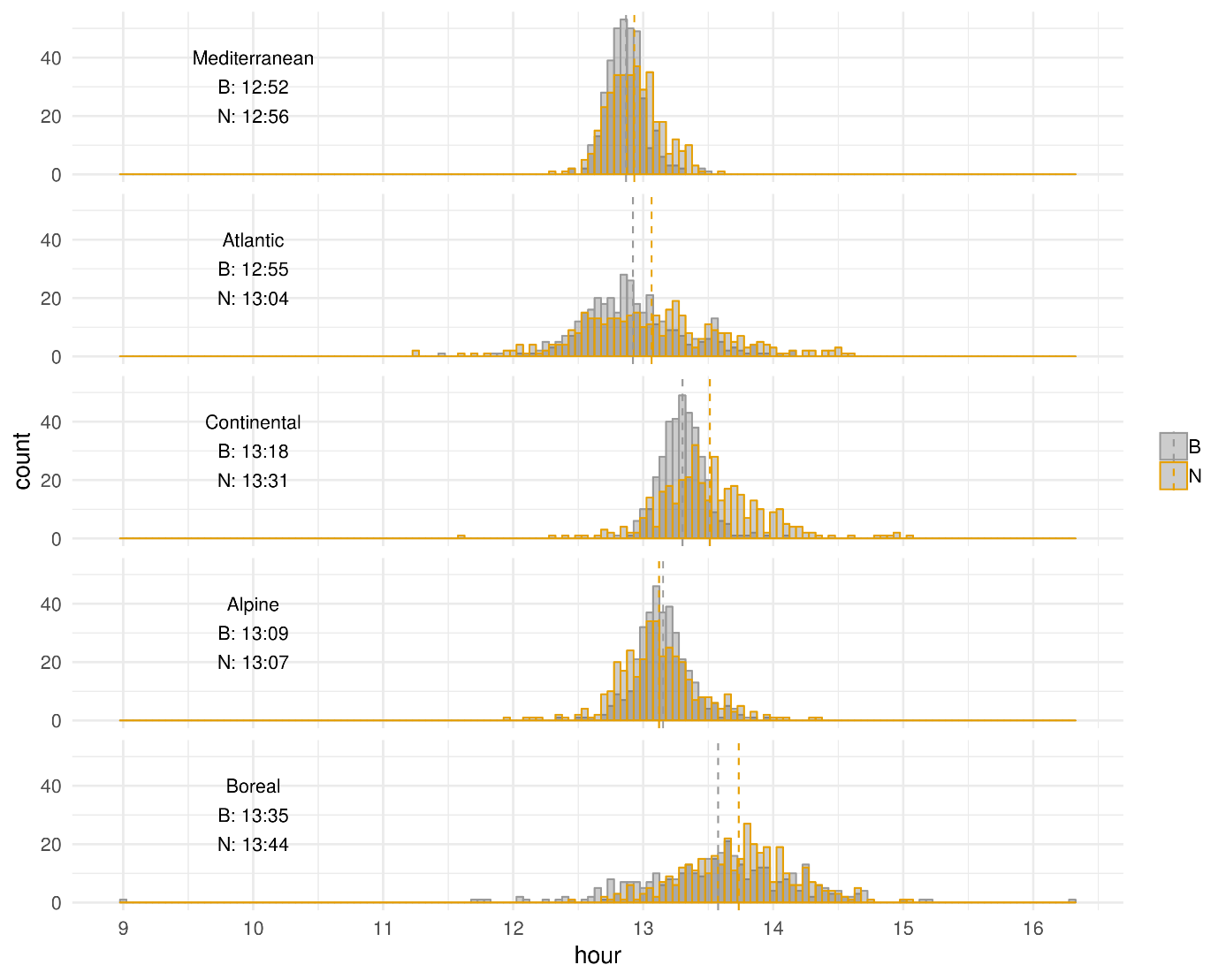


Figure S 3: Mean diurnal and seasonal cycles of 2m air temperature differences between broad-leaved and needle-leaved sites in Switzerland. Boxplots for each hour of the day indicate the spread of the observations and colored dots depict the median of the temperature difference.
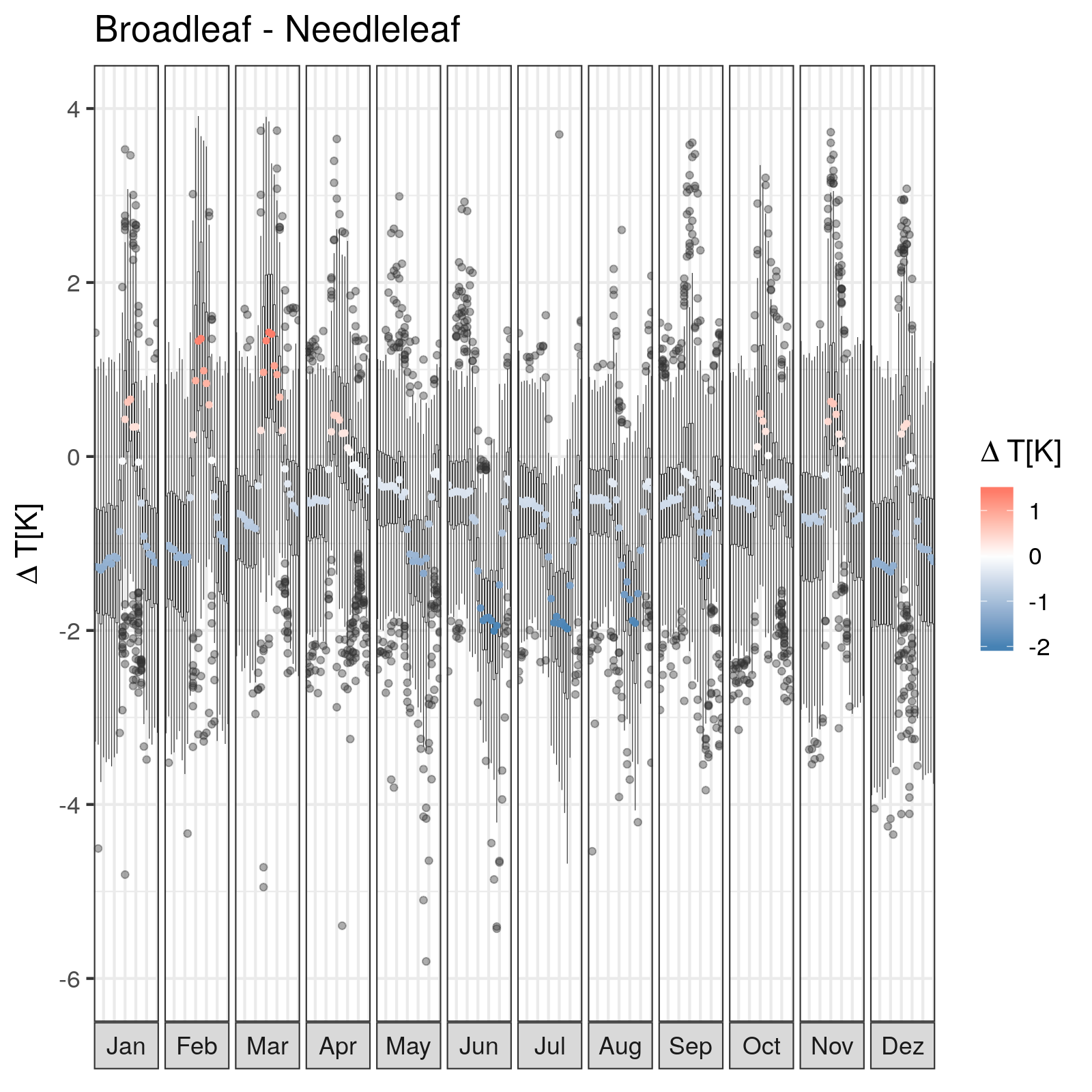


Figure S 4: Mean 2m air temperature differences in JJA between broad-leaved forest sites and needle-leaved forest sites. Error bars denote the standard deviation of the calculated differences.


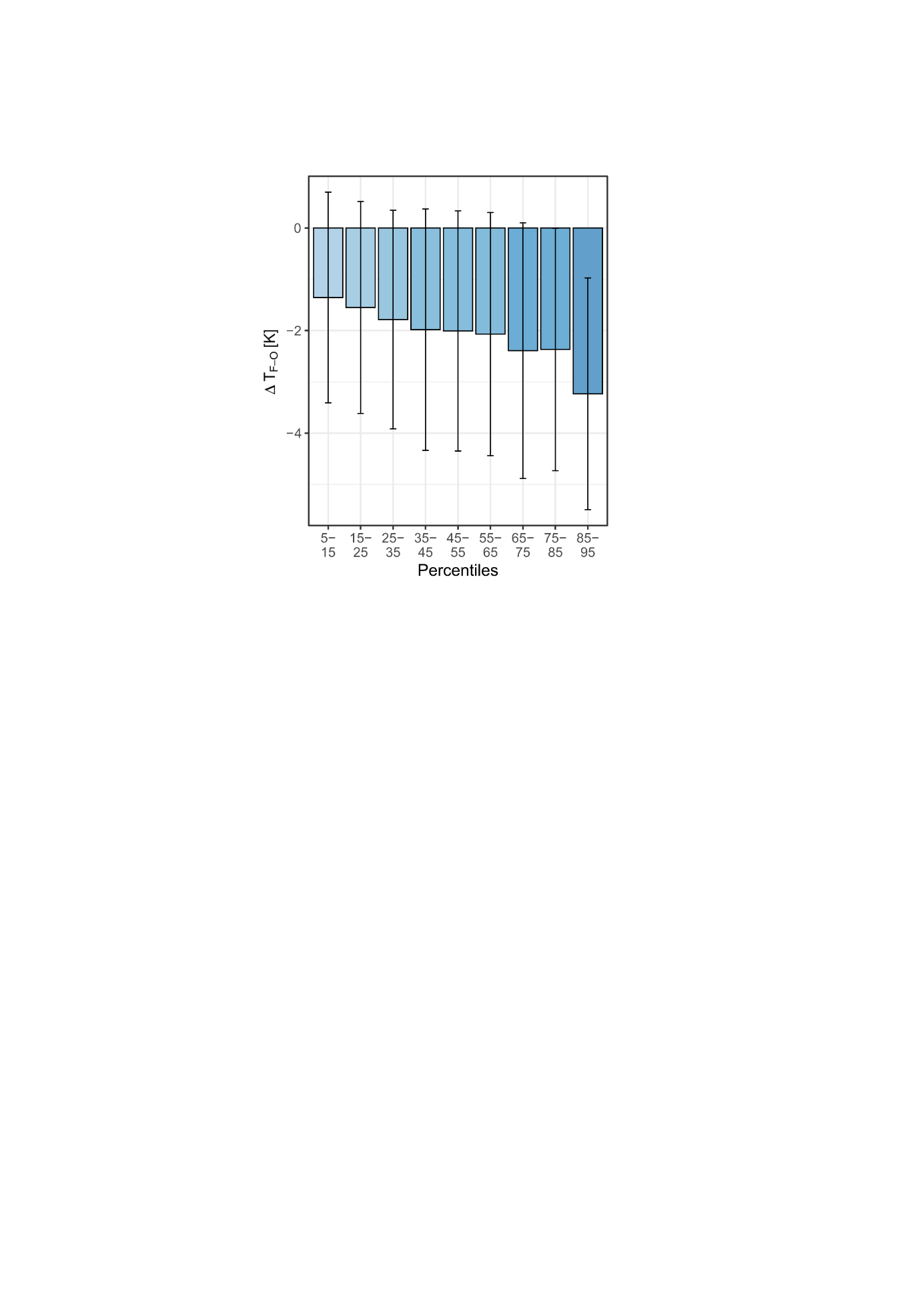


Figure S 5: Partial residuals and corresponding smoothing spline. The fraction of broad-leaved trees ranges from dark green (low fraction) towards light green (high fraction).


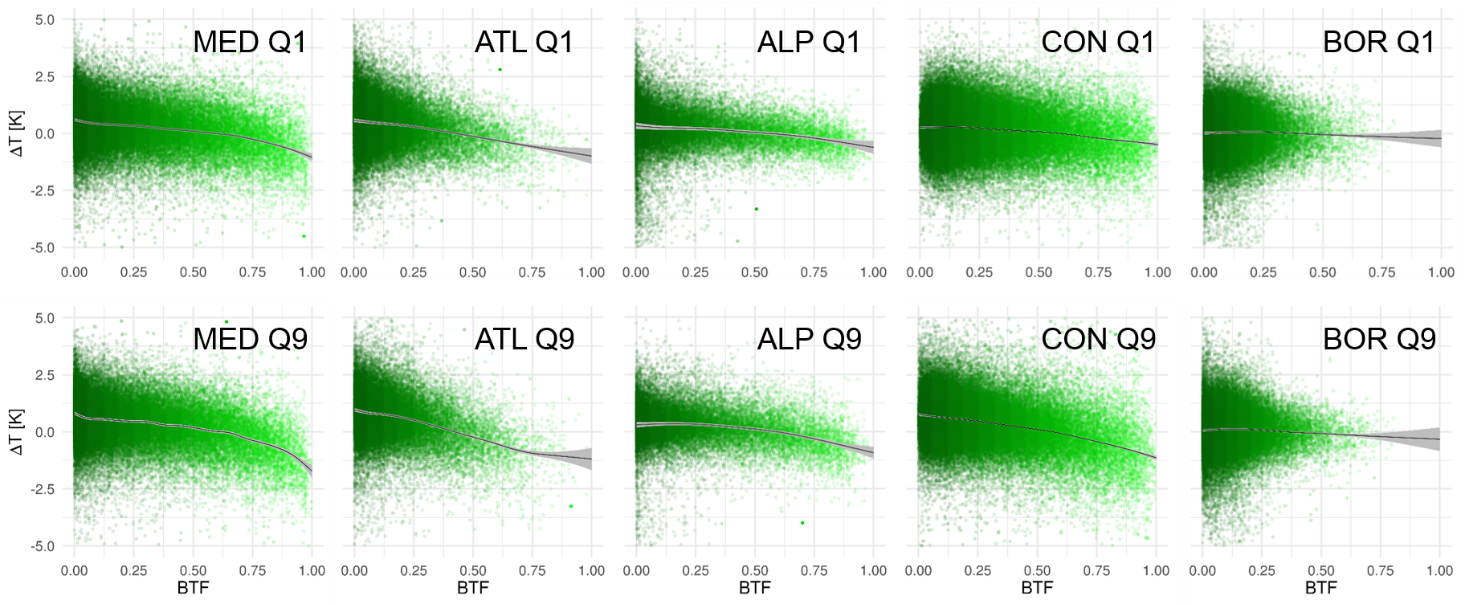


Figure S 6: Predictor variables describing the BTF and topographic properties.


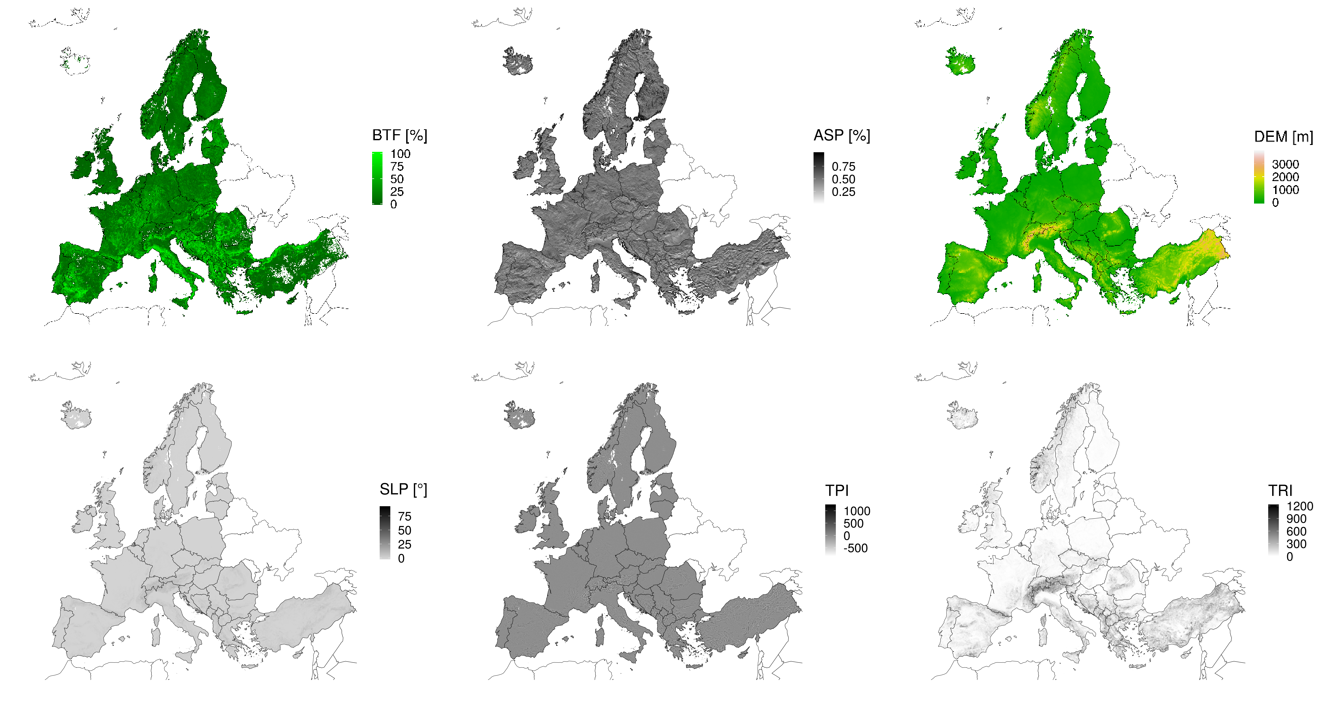


Figure S 7: Response variables used to estimate the BTF effect on temperature for different background climate conditions (here the different temperature quantiles for SEVIRI LST are shown).


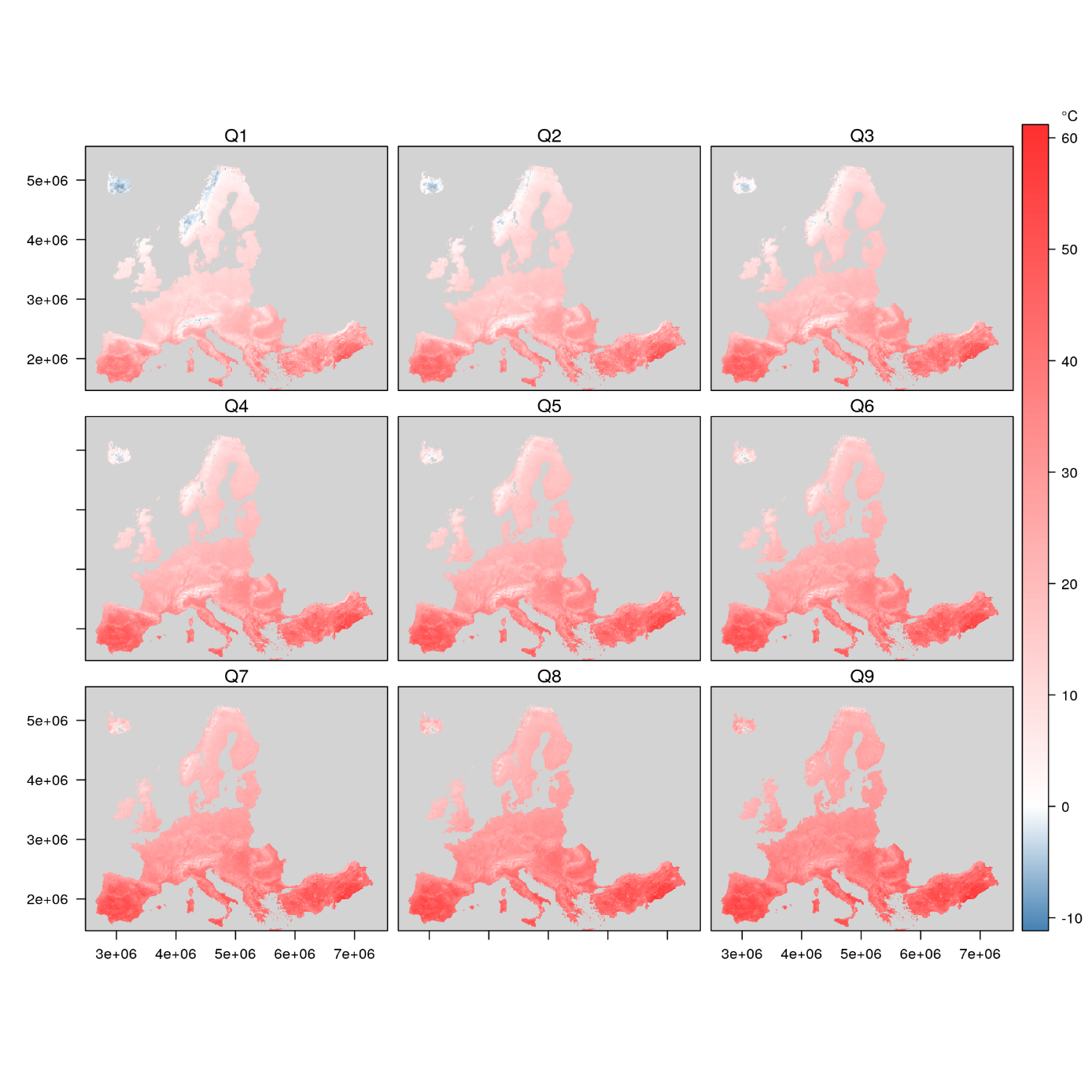


Figure S 8: Sensitivity of the effect of an increasing BTF when changing the basis dimension of the tensor product smooth interaction of the geographical coordinates x and y.


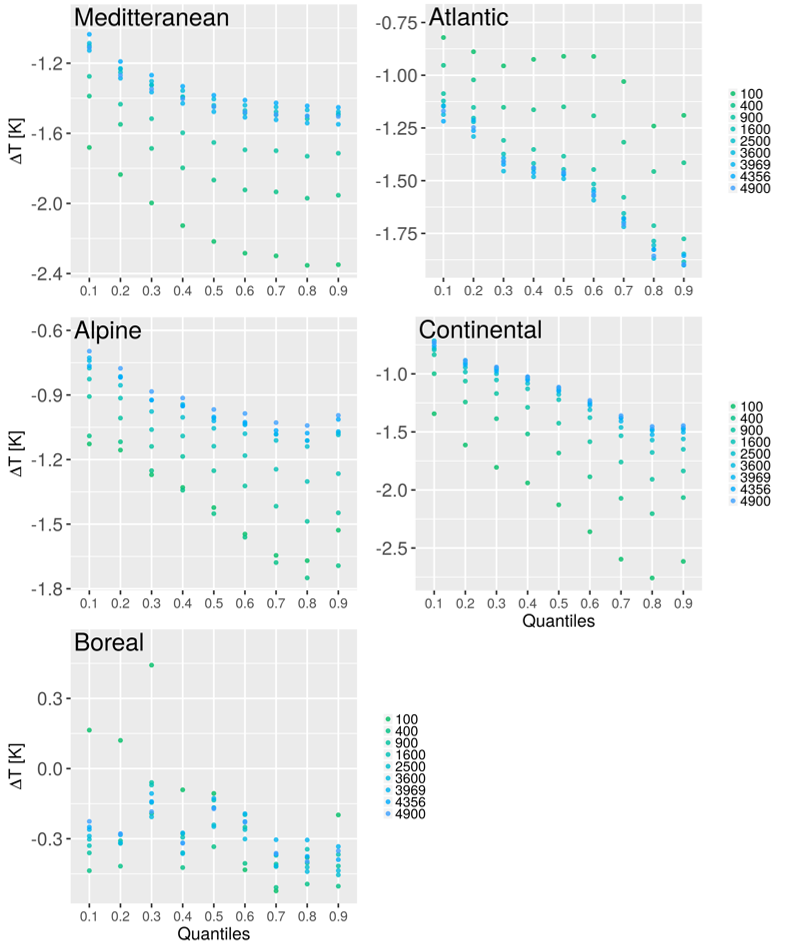


Figure S 9: Sensitivity of different models. Model E is the reference model that was used as the final model including all terms. Models A – D are modified versions of the original model. Model A: Reference models without any topographic predictor variables (DEM, SLP, ASP, TRI and TPI were excluded). Model B: Reference model, but SLP and TRI have been removed. Model C: Reference model, but the land cover data including 15 categories was replaced with data on 5 categories (Table S 2). Model D: Reference model without any information on land cover (i.e. only topographic variables included).


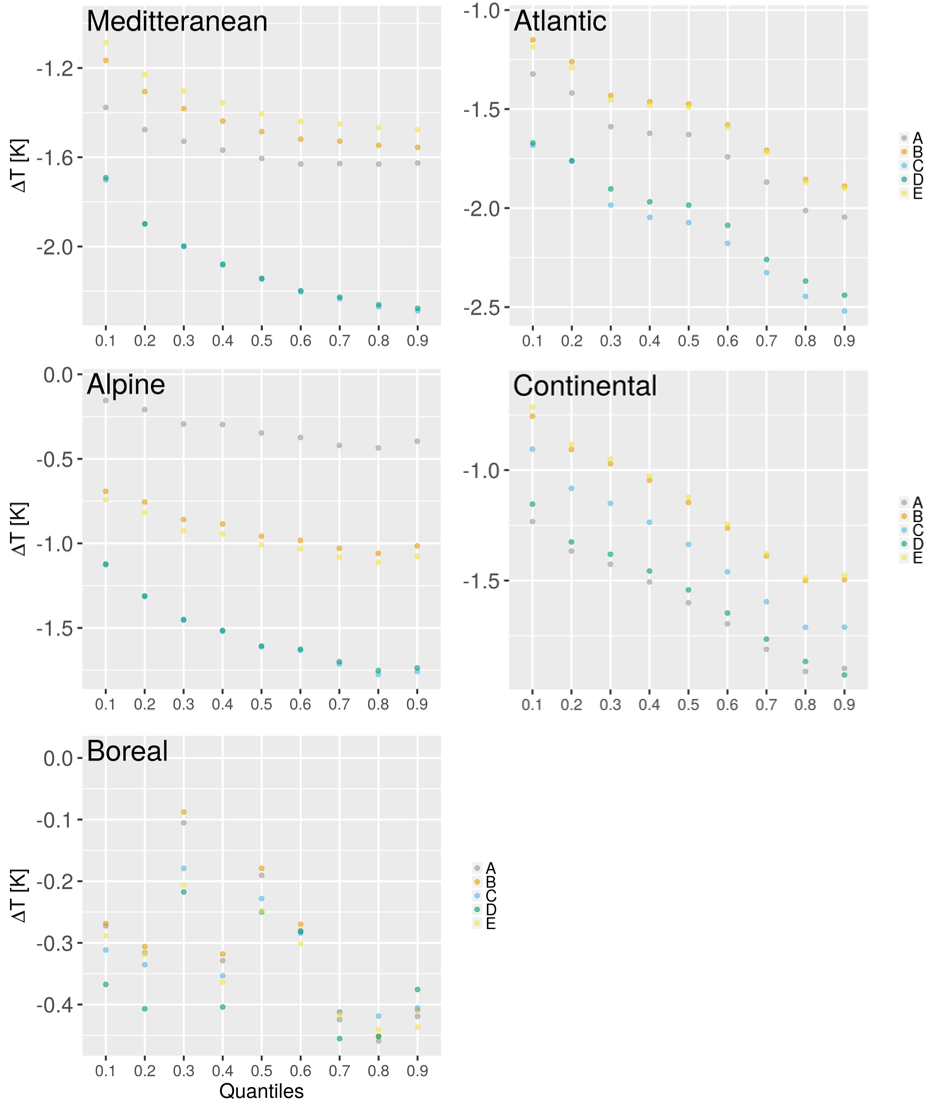


Figure S 10: Fraction of changes in forest type between 2012 and 2015. Increases in broad-leaved tree fraction from 2012 to 2015 are indicated as positive values and decreases as negative values.


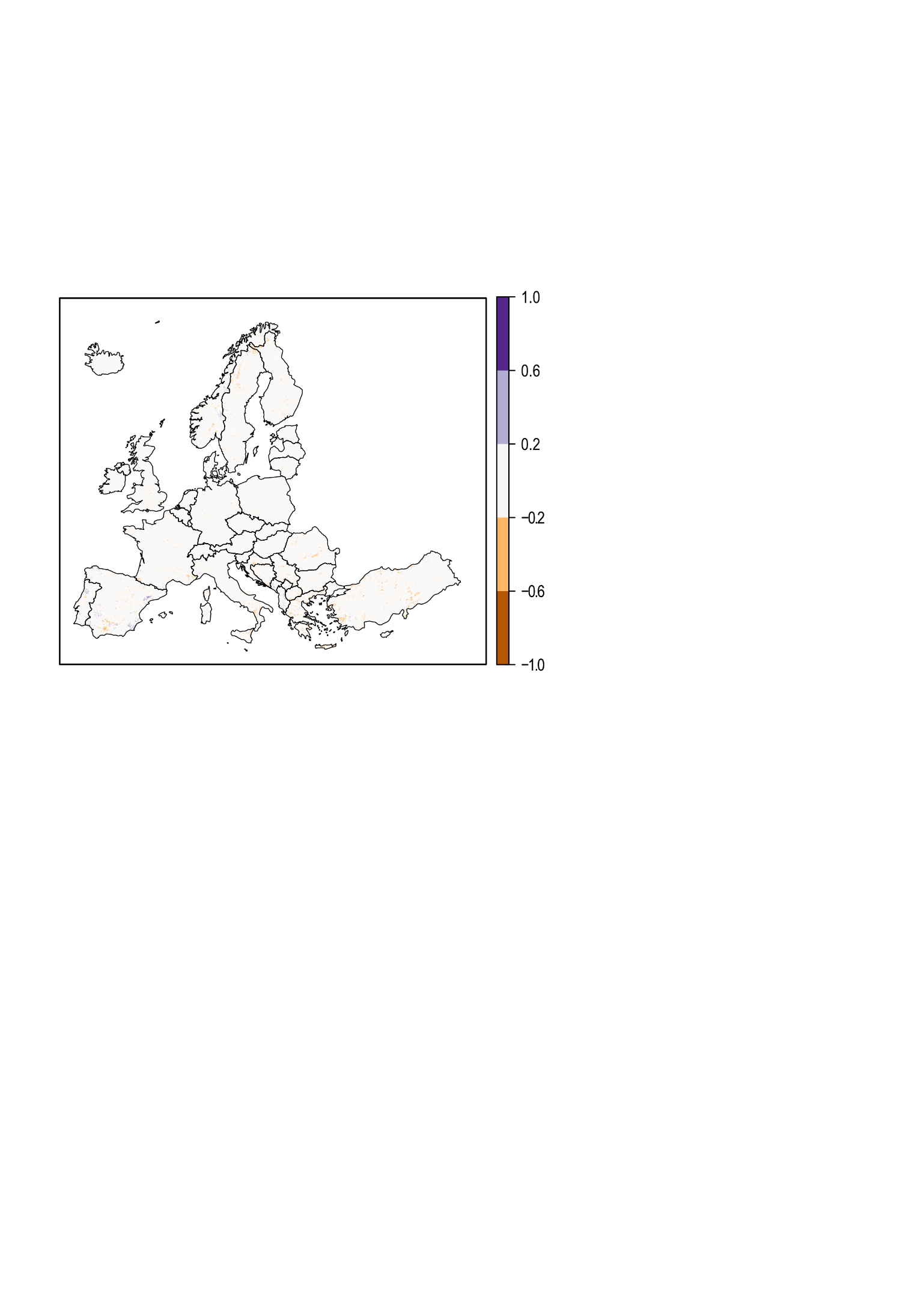


Figure S 11: Conversions involving three different forest types (broad-leaved, coniferous (i.e. needle-leaved) and mixed forest) for the two periods from 2006 to 2012 and 2012 to 2018 based on Corine land cover data.


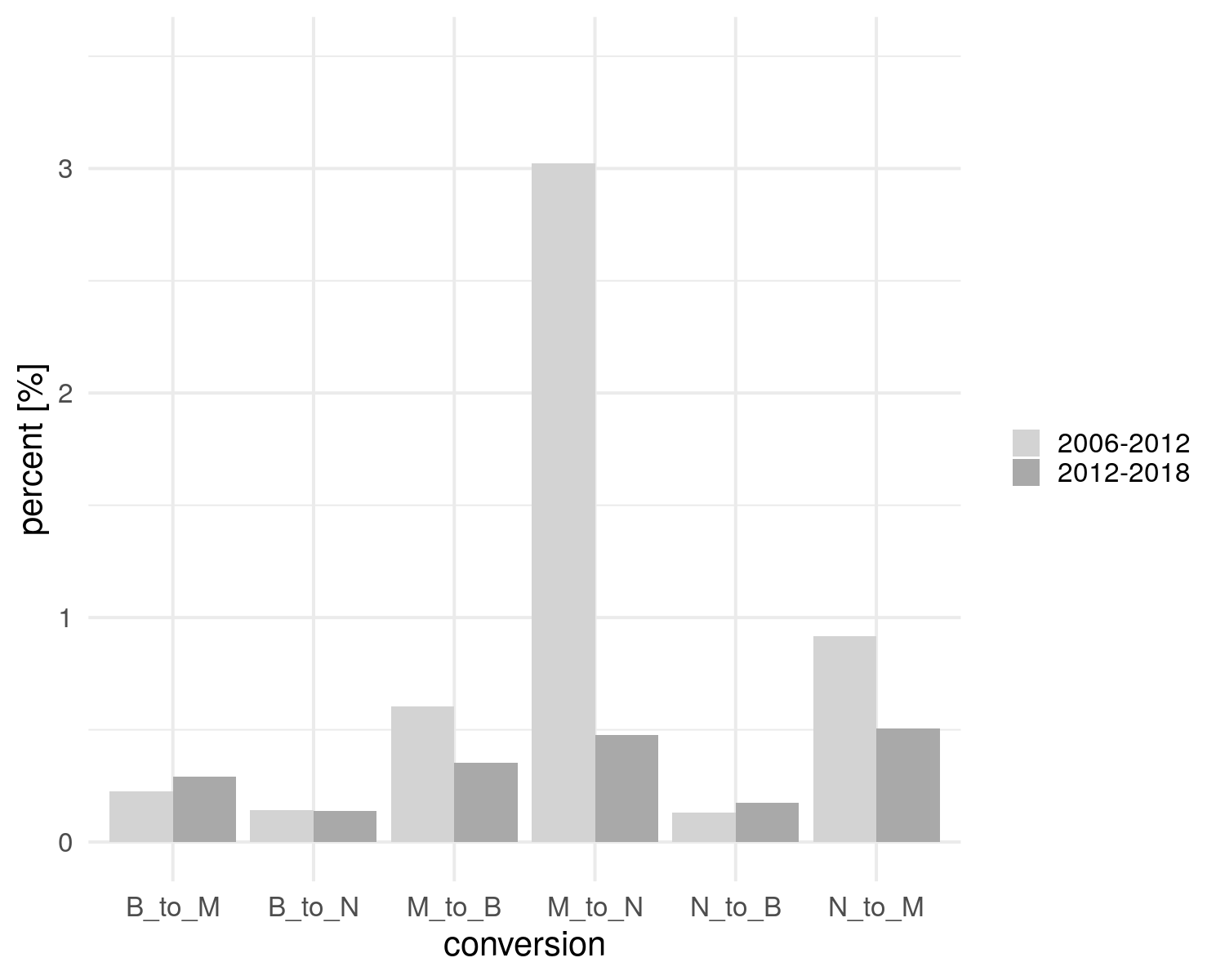


Table S 1: Response (bold font) and predictor variables.

| Variables | Temporal coverage and resolution | Spatial resolution (unmodified) | Source |
| --- | --- | --- | --- |
| **MODIS-LST (MLST)** | **01/01/2009 - 31/12/2014 (daily)** | **~1km** | **NASA** |
| **SEVIRI-LST (SLST)** | **01/01/2009 - 31/12/2014 (15 minutes)** | **~3-5km** | **EUMETSAT** |
| Broad-leaved Tree Fraction (BTF) | 2012 | 20m | Copernicus Land |
| Y-coordinates (latitude) | - | Inherent to MLST & SLST | Inherent to MLST/SLST |
| X-coordinates (longitude) | - | Inherent to MLST & SLST | Inherent to MLST/SLST |
| Elevation (DEM) | - | 100m | Copernicus |
| Slope (SLP) | - | 100m | (based on DEM) |
| Aspect (ASP) – fraction of north facing slopes | - | 100m | (based on SLP) |
| Terrain Ruggedness Index (TRI) | - | 100m | (based on DEM) |
| Topographic Position Index (TPI) | - | 100m | (based on DEM) |
| Land use/Land cover (LULC), transformed | 2012 | 100m | Copernicus (CORINE land cover) |

Table S 2: Three different levels of the Corine Land Cover classification system including 44, 15 and 5 categories.

| Land use/Land cover (LULC) |  |  |
| --- | --- | --- |
| 44 categories | 15 categories | 5 categories |
| 111 Continuous urban fabric, 112 Discontinuous urban fabric | 1 Urban fabric | 1 Artificial Surfaces |
| 121 Industrial or commercial units , 122 Road and rail networks and associated land , 123 Port areas , 124 Airports | 2 Industrial, commercial and transport units |  |
| 131 Mineral extraction sites, 132 Dump sites, 133 Construction sites | 3 Mine, dump and construction sites |  |
| 141 Green urban areas, 142 Sport and leisure facilities | 4 Artificial, non-agricultural vegetated areas |  |
| 211 Non-irrigated arable land, 212 Permanently irrigated land, 213 Rice fields | 5 Arable land | 2 Agricultural areas |
| 221 Vineyards,  222 Fruit trees and berry plantations, 223 Olive groves | 6 Permanent crops |  |
| 231 Pastures | 7 Pastures |  |
| 241 Annual crops associated with permanent crops, 242 Complex cultivation patterns, 243 Land principally occupied by agriculture, with significant areas of natural vegetation,  244 Agro-forestry areas | 8 Heterogeneous agricultural areas |  |
| 311 Broad-leaved forest, 312 Coniferous forest, 313 Mixed forest | 9 Forests | 3 Forests and semi natural areas |
| 321 Natural grasslands, 322 Moors and heathland, 323 Sclerophyllous vegetation, 324 Transitional woodland-shrub | 10 Scrub and/or herbaceous vegetation associations |  |
| 331 Beaches, dunes, sands, 332 Bare rocks, 333 Sparsely vegetated areas, 334 Burnt areas, 335 Glaciers and perpetual snow | 11 Open spaces with little or no vegetation |  |
| 411 Inland marshes, 412 Peat bogs | 12 Inland wetlands | 4 Wetlands |
| 421 Salt marshes, 422 Salines, 423 Intertidal flats | 13 Maritime wetlands |  |
| 511 Water courses, 512 Water bodies | 14 Inland waters | 5 Water bodies |
| 521 Coastal lagoons, 522 Estuaries, 523 Sea and ocean | 15 Marine waters |  |

Supplement S 1: Study domain.

While SEVIRI LST and MODIS LST are available on a large and MODIS even on a global scale (as well as most predictor variables), our study domain is constrained by the information about the broad-leaved tree fraction BTF in the European Union. The BTF data is only available within the administrative boundaries of the following countries: *Albania, Austria, Bosnia and Herzegovina, Belgium, Bulgaria, Switzerland, Cyprus, Czech Republic, Germany, Denmark, Estonia, Spain (including Andorra), Finland, France, United Kingdom, Greece, Croatia, Hungary, Ireland, Isle of Man, Iceland, Italy, Liechtenstein, Lithuania, Luxembourg, Latvia, Montenegro, Republic of North Macedonia, Martinique, Malta, Northern Ireland, Netherlands, Norway, Poland, Portugal, Romania, Serbia, Sweden, Slovenia, Slovakia, Turkey, Kosovo*

Supplement S 2: Transformation of the land cover data before including it into the Generalized Additive Models.

All land-cover data was transformed before it was included into the model, because it is an inherent property of the land cover data that each land-cover category is a relative proportion and all categories sum up to 100% (e.g. the area of a pixel may be covered by 15% agricultural land and 85% forest). This type of data is referred to as compositional data and requires careful treatment, one reason being that the single components (i.e. proportions of land-covers) may not be linearly independent [[1](#_ENREF_1)]. We transformed the land cover data using an isometric log-ratio transformation following [van den Boogaart and Tolosana-Delgado [2]](#_ENREF_2). As a first step, this involved a centered log-ratio transformation (clr), which can be described as:

|  | $clr(x)=\left( ln\frac{x_{i}}{g(x)} \right)_{i=1,\ldots,D} with g\left( x \right)= \left( \prod_{j=1}^{D} x_{j} \right)^{1/D}$ | 3 |
| --- | --- | --- |

where the divisor $g(x)$ is the geometric mean of the $D$ components (i.e. the percentages of each land-cover category within a pixel) of the composition. The isometric log-transformation is then given by

|  | $\mathrm{ilr}\left( x \right)=V^{t}\mathrm{clr}\left( x \right)$ | 4 |
| --- | --- | --- |

with $V$ being a matrix which columns form an orthonormal basis of the clr-plane. In this case $V$ is obtained by removing the first row from the so-called Helmert matrix. All transformations were carried out using the R package “compositions” [[3](#_ENREF_3)]

Supplement S 3: Additional discussion and validation of the models.

**Validation and uncertainties.** The average R2 when fitting models for each quantile was above 0.8 for all regions. In the Mediterranean, Atlantic and Continental they were above 0.9 and hence slightly higher than in the Boreal and the Alpine region. The models fitted to reflect the mean diurnal and seasonal cycles also showed a decent fit (R2 again above 0.8). Residuals were checked for each model (relying on the function gam.check in the R package mgcv) and are shown exemplarily for quantile 0.5 and the atlantic region (Figure S 12). The scaled residuals have approximately constant variance for all models in the different regions. The QQ-plots show that there is some deviation from the theoretical quantiles. However, this is of minor importance for our analysis, since we do not perform statistical tests that rely heavily on assumptions about the distribution of the residuals.

All models were fitted relying on thin plate regression splines and tensor product smooths. To check that the basis dimension for the smoothers was not restrictively low, we plotted the partial residuals against the term estimates looking for systematic patterns in the residuals that were not captured by the smoother. There was no indication of such patterns. However, the scale parameters obtained when differencing residuals that are neighbors according to the x and y coordinates are sometimes significantly different from scale parameter estimates of randomly ordered residuals. This indicates that the basis dimension for this smooth (tensor product smooth of the interaction of x and y coordinates) might be too low. To further check, whether the basis dimension for this tensor product smooth was too low, we refitted the model with increased basis dimension for this smooth. However, between a basis dimension of 1600 and 3600 (i.e. k=40 and k=60), there was neither much change in the results according to the effect of an increased fraction of broad-leaved trees, nor did we find a pattern when fitting a model using the deviance residuals as a response and x and y as covariates (i.e. predictors).

Models fitted for certain regions (in particular Boreal and Continental) and models for which we included an interaction between BTF and the x and y coordinates showed sometimes high values of pairwise concurvity between the BTF smooth and other smooths (Figure S 13). Concurvity can be roughly described as nonlinear dependencies among predictor variables. It is sometimes considered as a generalization of co-linearity and can cause similar issues. Pairwise concurvity estimates between the BTF smooth and other terms of the model are often high for the variables SLP (Slope) and TRI (Terrain Ruggedness Index) and for some of the transformed land cover variables. Unsurprisingly, concurvity sometimes also occurs to be large between the BTF smooth and the spatial coordinates smooth. To test how sensitive our results are if the model structure is changed we fitted several additional models without including certain terms (e.g., SLP and TRI), by using a different land cover scheme (i.e. a classification system of only 5 different land cover classes) and by testing the sensitivity of a varying basis dimension of the tensor product smooth interaction of x and y (i.e. the spatial coordinates smooth). These results are qualitatively very similar to our results, but there are some differences (Figure S 8 and Figure S 9). Beyond a basis dimension of 1600 the potential effect of an increased BTF on temperature does not change substantially. Increasing the basis dimension in the Mediterranean, Alpine and Continental region decreases the absolute value of the temperature response, whereas it increases the absolute value in the Atlantic. However, this trend can only be observed for small basis dimensions (< 1600). For large basis dimensions there is no clear trend of an increase or decrease (Figure S 8). Removing all topographic predictors from the model has a strong effect on the potential change in temperature in the Alpine and the Continental region. Removing all predictors on land cover or replacing the rather detailed land cover information including 15 categories with information on only 5 categories has a pronounced effect in almost all regions. The effect of an increased BTF on temperature tends to be more negative when only reduced or no information on land-cover is included.

The analysis of in-situ observations corroborates the findings obtained from remote sensing observations. However, besides some of the already mentioned limitations it should also be noted that the forest sites analyzed are characterized by different broad-leaved and needle-leaved species. These include three European beech (Fagus sylvatica), two oak (Quercus robur and Quercus cerris), two Norway spruce (Picea abies), one Mountain pine (Pinus mugo), one Scots pine (Pinus sylvestris) and one mixed Larch/Arolla pine site (Larix decidua/Pinus cembra). Some of the species are most likely over- or under-represented considering how much of the forested area in Switzerland or the entire Alpine region is actually covered by them. This is relevant since, e.g., the temperature differences between beech and the two needle-leaved species Mountain pine and Scots pine are very large and the differences between Beech and Larch/Arolla pine are rather small [[4](#_ENREF_4)]. This could also be the reason why the cooling signal in summer is higher for the in-situ observations (Figure 2, Figure S 2).

High uncertainties of the temperature signals in the Boreal compared to other regions, as indicated by large confidence intervals (Figure 1), are related to the relatively sparse occurrence of broad-leaved forests in this region. In particular, areas covered by high fractions of broad-leaved trees (> 60%) are scarce and hence statistical predictions on the temperature difference between broad- and needle-leaved forests involve larger uncertainties. In addition, LST data derived from the SEVIRI sensor have higher uncertainty due to larger viewing angles of the geostationary satellite [[5](#_ENREF_5)].

We assume that the broad-leaved tree fraction and land cover from 2012 remains largely constant within the study period between the beginning of 2009 and the end of 2014. In the Mediterranean and the Boreal region there are some noticeable changes in the broad-leaved tree fraction (Figure S 10). In addition, there were transitions from mixed to needle-leaved forest between 2006 and 2012 (Figure S 11). Since the amount of land cover changes were relatively small, they are likely not having a bigger impact on the results obtained for the different regions. However, these changes may be very relevant when considering more localized temperature changes caused by an increase in the broad-leaved fraction as also shown (**Error! Reference source not found.**).

**Discrepancies between SEVIRI and MODIS-LST.** We showed that the mean temperature response of an increase in BTF can be approximated using only two LST observations per day as being available from MODIS (onboard the Aqua satellite, Figure S 1). However, adding data on high temporal resolution fills a gap in the analysis of biophysical climate impacts of LULUCF. It allows to better assess diurnal cycles of temperature effects and the effects during hot extremes. Thus, it could be very relevant to employ such high temporal resolution data not only concerning an increasing BTF, but also concerning the analysis of any other type of land management and land cover change.

Although the results obtained relying on MODIS and SEVIRI LST do in general agree well, there are some noticeable discrepancies. During summer in the Mediterranean, MODIS exhibits a moderate signal comparison than SEVIRI. One reason for the discrepancy could be that both datasets rely on different assumptions about emissivity and on a more or less restrictive cloud-masking [[6](#_ENREF_6), [7](#_ENREF_7)]. For example, the SEVIRI LST algorithm uses a dynamic emissivity map, i.e., emissivity is altered according to the observed vegetation state (Trigo et al., 2008). MODIS emissivities are classification-based (Wan, 2014) and may hence not always capture different states of the vegetation (e.g. the influence of droughts). The discrepancy in the boreal region could be caused by different viewing geometries. SEVIRI scans the boreal regions with a large viewing angle. LST retrievals at large viewing angles have significant uncertainties [[8](#_ENREF_8)]. The LST derived from the nadir looking MODIS instrument is not effected by those uncertainties. LST retrievals over mountain areas are subject to systematic errors caused by viewing effects and uncertainties in the atmospheric characterization [[9](#_ENREF_9)]. Hence, differences between the nadir looking MODIS and the side viewing SEVIRI instrument in the alpine region are also to be expected. In addition, differences in the results between MODIS and SEVIRI may originate from the different resolutions of the two datasets. These can lead to a change in the covariance structure of the data and hence differences in the estimated magnitude of the BTF effect. Although the higher spatial resolution of MODIS is generally favorable, the results obtained from MODIS and SEVIRI overall agree very well. This indicates that the spatial resolution of SEVIRI is sufficiently high to adequately capture the effect of different forest types on land surface temperatures.**Comparison with previous findings.** The temperature response of an increasing BTF has not yet been assessed systematically in many observational studies. Nevertheless, there are quantitative studies which allow a comparison with our results. A potential cooling effect of broad-leaved trees has also been reported by [Renaud, Innes [4]](#_ENREF_4) relying on station observations in Switzerland. Their study shows that temperature change related to an increase in BTF may not only exist for land surface but also 2m air temperature. In agreement with our results they also show that differences in temperature between broad-leaved and needle-leaved sites tend to increase during hot temperatures. Based on satellite observations, [Zaitchik, Macalady [10]](#_ENREF_10) show that the 2003 heat wave in France was less severe for coniferous forests (exhibiting lower temperatures) than for deciduous broad-leaved forests. However, they mention that this difference can be partly explained because in their study domain coniferous forests were found at higher altitude and on north-facing slopes, which highlights the importance of accounting for these potential confounding factors. The impact of an increase in BTF on seasonal temperatures was also assessed based on a dataset provided by [Duveiller, Hooker [1]](#_ENREF_1), showing cooling in summer in the Mediterranean and Atlantic region, but some warming in the Continental and Alpine region. However, a comparison with this dataset is only possible in a limited manner, since [Duveiller, Hooker [1]](#_ENREF_1) masked, e.g., regions with high topographic gradients.

So far we know that the temperature differences between broad-leaf and needle-leaf forests during summer days might be related among other things to a larger fraction of shortwave radiation being reflected (i.e. a higher albedo) and larger latent heat fluxes over broad-leaf forests due to larger stomatal conductance [[11-14](#_ENREF_11)]. In addition, a higher interception observed for broad-leaf forests [[15](#_ENREF_15)] could lead to higher soil moisture and hence help to maintain higher evapotranspiration. However, there is hardly any observational evidence on how relevant each of these factors are. To better understand these factors would also help to predict how the temperature signal caused by an increase in BTF may change under future environmental conditions.

**Potential shortcomings and confounding factors.** We employed a space-for-time analogy to extract the BTF signal as in previous studies [cf. [14](#_ENREF_14), [16](#_ENREF_16)]. When relying on static data of forest characteristics, establishing a causal link between a change in BTF and the resulting effect on temperature has to be done very carefully. We have considered a larger number of predictor variables concerning topography and land cover to account for potential confounding factors in the statistical model. Nevertheless, some confounding effects cannot be totally ruled out. For example, broad-leaved forests might naturally occur more often at moist sites which can, e.g., be the case for dense forests [[17](#_ENREF_17)]. However, the difficulty in establishing a causal relationship is partly alleviated by the fact that historic forest management and disturbances caused by humans have likely led to patterns of broad-leaved versus needle-leaved forests that are independent of climatic site characteristics [[18](#_ENREF_18)]. It should also be noted that broad-leaved-rich forests tend to have a higher tree cover density than needle-leaved sites (Figure S 14). This is most likely related to the morphological differences between broad-leaved and needle-leaved trees [[e.g. 19](#_ENREF_19)], but we cannot exclude that in some cases it might also reflect different management practices at needle-leaved versus broad-leaved sites, which would be an important aspect to consider in future research.

Finally, analyzing temporal changes in forest cover and temperature can be a complementary approach [[20](#_ENREF_20)]. However, since shifts in forest composition (e.g. changes from needle-leaved to broad-leaved forests) are usually occurring over decades, current remote sensing time series are not very well suited for such an analysis. However, establishing causality may also be an issue in the temporal approaches due to the effect of other climate forcings and climate variability.

**Small scale spatial variation of the temperature signal related to an increase in BTF.** We did our main analysis for five different biogeographical regions assuming that they would allow us to detect major differences in the potential effect of an increased broad-leaved tree fraction in forests. Results from the additional analysis in which we allowed variation of the BTF at smaller scales, reveal that there may be additional patterns of the temperature change caused by an increase in BTF (Figure S 15). However, these patterns have to be interpreted carefully. Plotting the spatially explicit standard error against the temperature effect of an increased BTF shows that uncertainty strongly increases when results show a potential warming or strong cooling (i.e. large uncertainties roughly for ΔT < -4 K and ΔT > 0 K) related to an increase in BTF (Figure S 16). These uncertainties also strongly influence the aggregated effect in different regions (i.e. calculating mean values in each region, Figure S 17). For example, in the Atlantic region, the mean cooling effect of BTF calculated with all values differs substantially from the cooling effect calculated only from LST values with a standard error smaller than 0.5 K in (Figure S 17). In the latter case results become similar to the ones obtained when fitting models for each region instead of allowing for interaction between BTF and the spatial coordinates for whole Europe.

[_ENREF_29](#_ENREF_29)

Figure S 12: Model diagnostics for the Atlantic region and temperature quantile 0.5 as response variable.


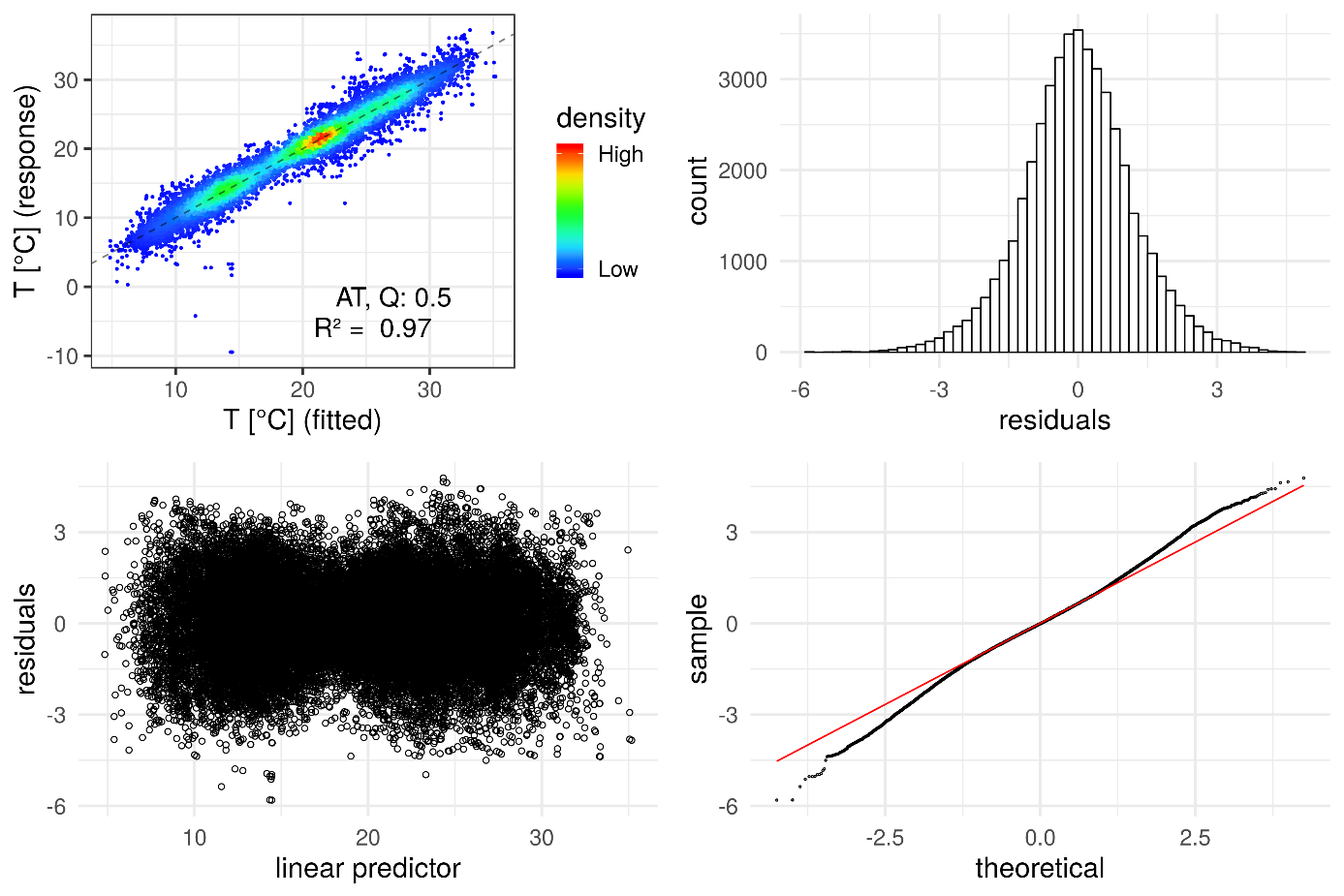


Figure S 13: Pairwise concurvity measure for every region. The left column (with five regions) is based on SEVIRI LST and temperature quantile 0.5 as response variable. The right column is based on MODIS LST and temperature quantile 0.5 as response variable. Visualization based on the function vis.concurvity.R (<https://gist.github.com/dill/c148e4629333212ec7be>).


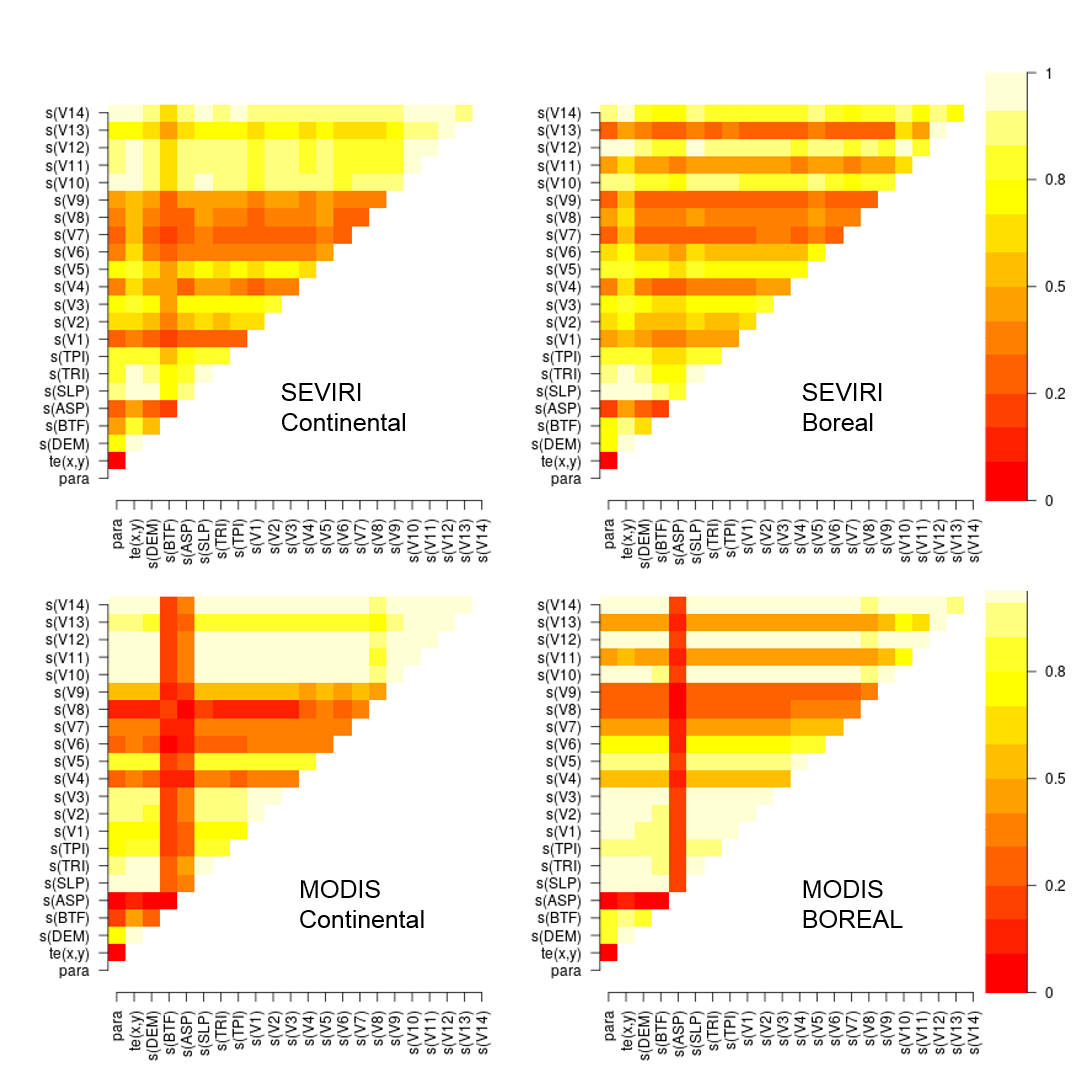


Figure S 14: Correlation between tree cover density (TCD) and broad-leaved tree fraction (BTF).


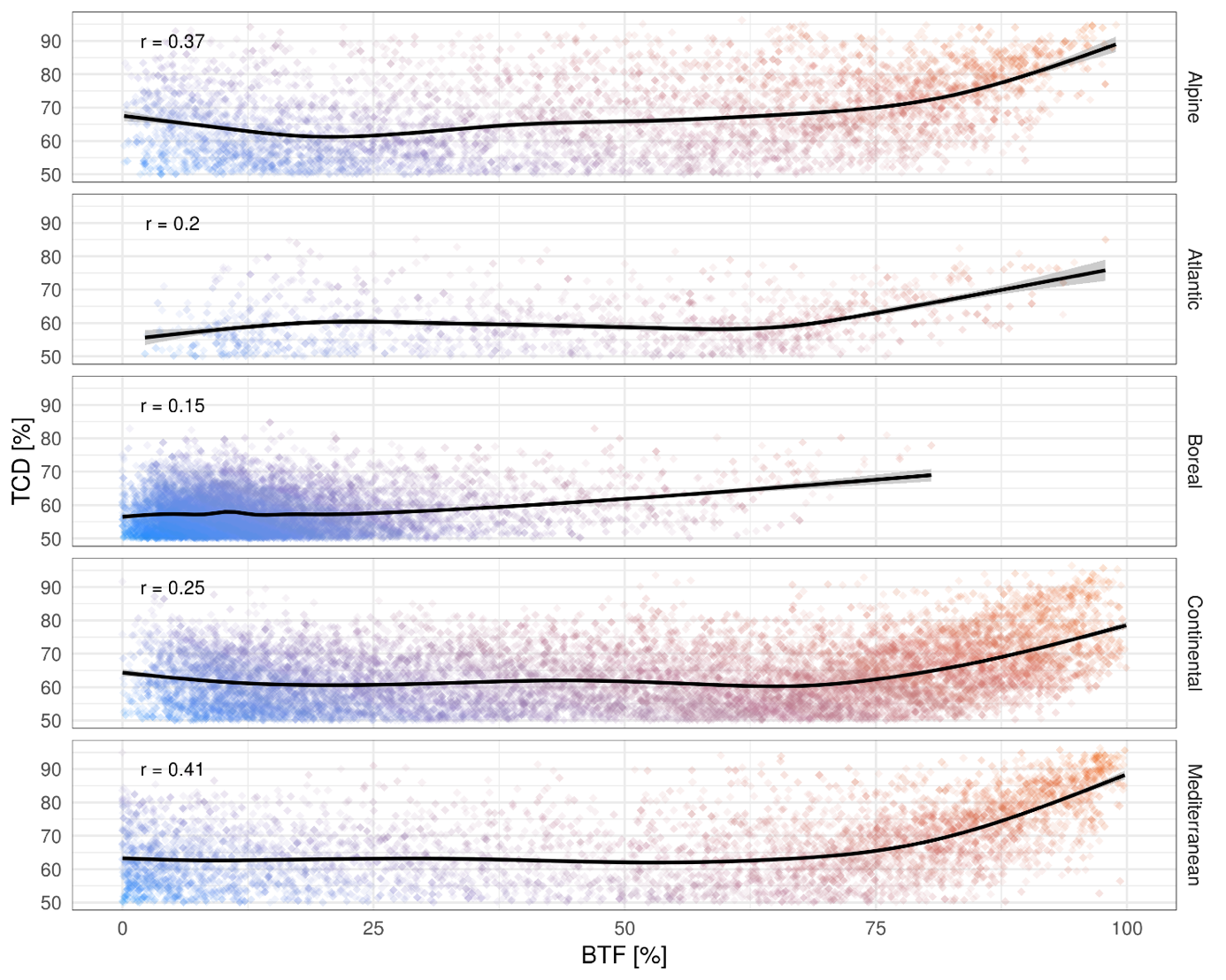


Figure S 15: Spatially varying effect of an increasing BTF on temperature for different SEVIRI LST quantiles. Grid cells showing a standard error larger than 2 K have been removed.


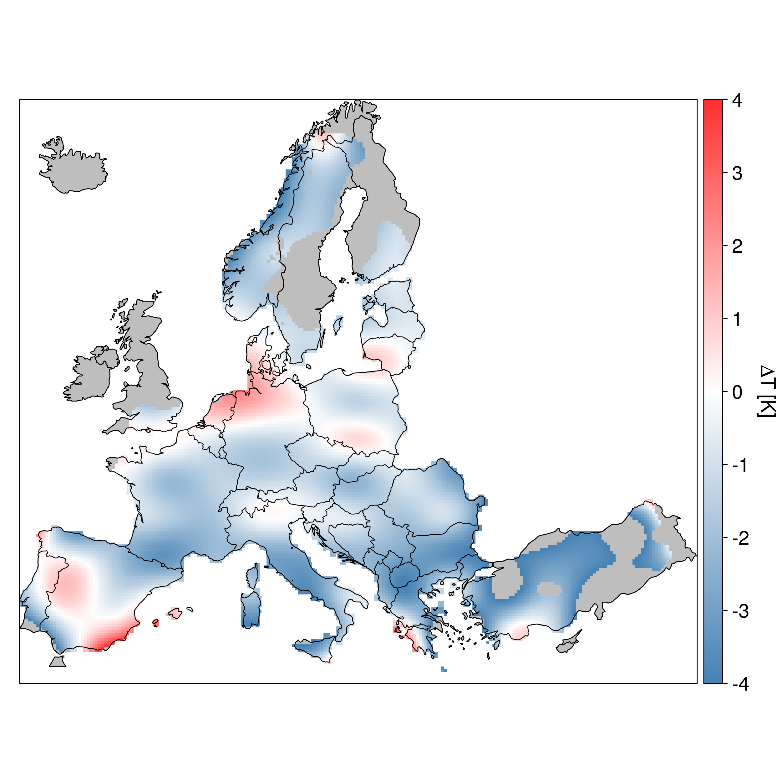


Figure S 16: Standard error of the potential change in temperature caused by an increase in BTF versus the potential change in temperature. Each point represents the value of a pixel.


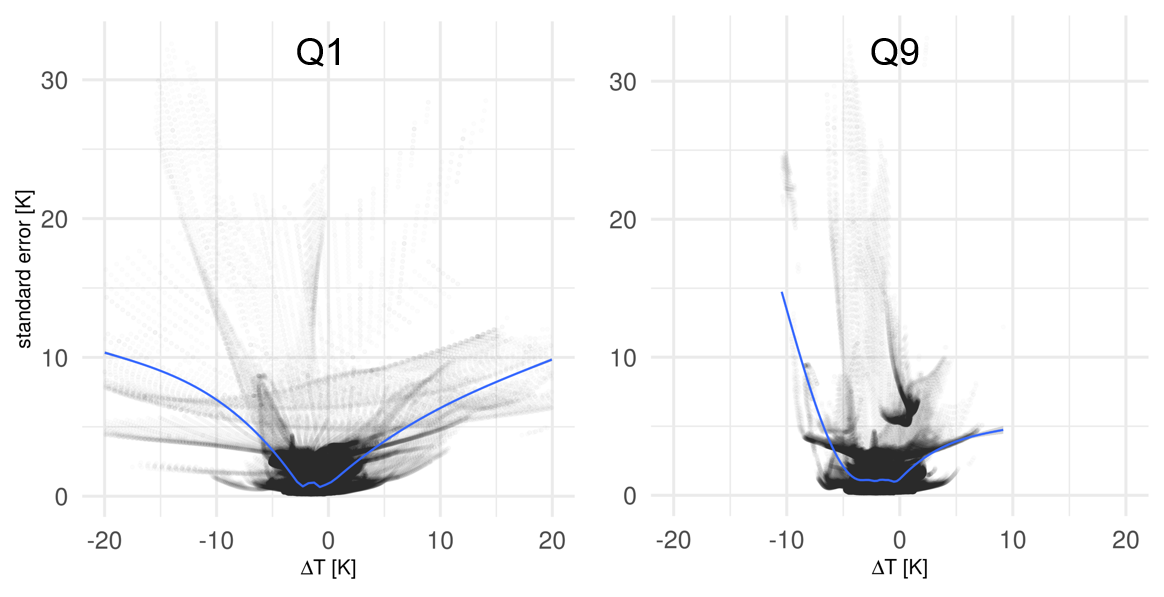


Figure S 17: The potential effect of an increasing BTF on temperature for five different regions aggregated based on the spatially explicit results (i.e. allowing for interaction between the spatial coordinates and BTF, Figure S 15). Left: Mean values calculated for each region including all values. Right: Mean values calculated for each region after pixels of a standard error larger than 0.5 have been removed. Since all values in the boreal region exhibit errors larger than 0.5 there is no mean value in this region.


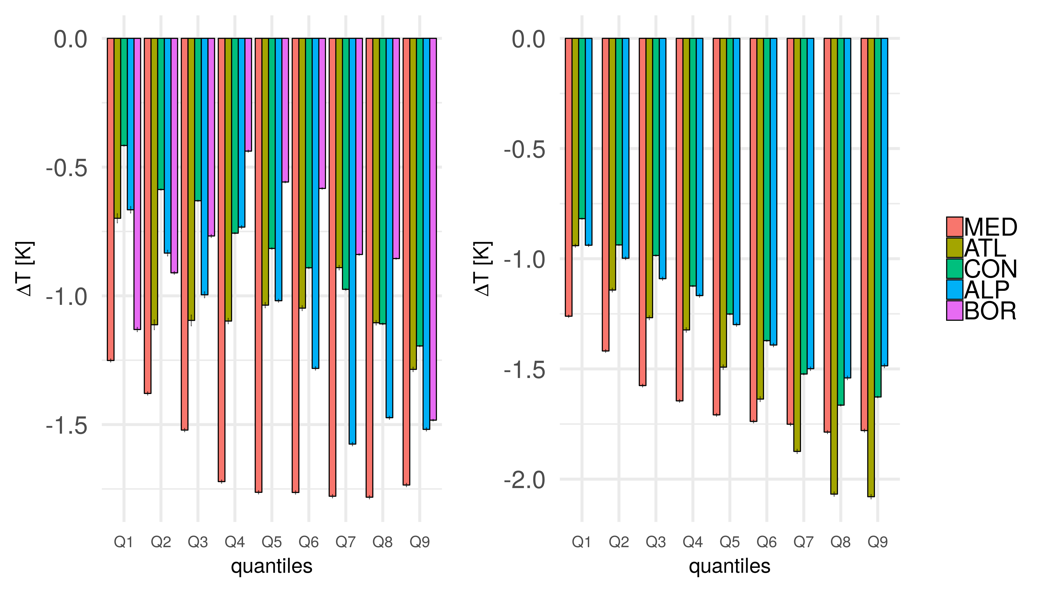


Table S 3: Adapted from [[21](#_ENREF_21)]. Summary of characteristics of each paired site.

| Site name | Forest type | Altitude [masl] | Orientation forest/open | LAI | Distance | latitude forest/ open | longitude forest/ open | Broad-leaved, Needle-leaved |
| --- | --- | --- | --- | --- | --- | --- | --- | --- |
| Beatenberg | Picea abies | 1523 | S/S | 1.9 | 666 | 7.76392/  7.77215 | 46.6999/  46.7004 | N |
| Chironico | Picea abies | 1381 | N/E | 3.7 | 729 | 8.81281/  8.81822 | 46.4462/  46.4408 | N |
| Celerina | Larix decidua, Pinus cembra | 1866 | NE/Slope < 5 | 1.2 | 1787 | 9.88881/  9.87587 | 46.4921/  46.5060 | N |
| Isone | Fagus sylvatica | 1194 | N/NE | 5.8 | 145 | 9.00769/  9.00731 | 46.1250/  46.1260 | B |
| Jussy | Quercus robur | 497 | None/Slope < 5 | 5.8 | 571 | 6.28694/  6.29315 | 46.2284/  46.2312 | B |
| National_park | Pinus mugo | 1899 | S/S | 1.3 | 924 | 10.2304/  10.24102 | 46.6616/  46.6596 | N |
| Neunkirch | Fagus sylvatica | 562 | N/Slope < 5 | 5.2 | 906 | 8.53455/  8.52635 | 47.6840/  47.6898 | B |
| Novaggio | Quercus cerris | 929 | S/S | 3.8 | 273 | 8.83428/  8.83519 | 46.0216/  46.0241 | B |
| Othmarsingen | Fagus sylvatica | 475 | S/S | 4.6 | 343 | 8.22631/  8.22289 | 47.3985/  47.4002 | B |
| Visp | Pinus sylvestris | 698 | N/Slope < 5 | 2.3 | 1287 | 7.85708/  7.85828 | 46.2969/  46.2981 | N |

1. Duveiller, G., J. Hooker, and A. Cescatti, *A dataset mapping the potential biophysical effects of vegetation cover change.* Scientific Data, 2018. **5**: p. 180014.

2. van den Boogaart, K.G. and R. Tolosana-Delgado, *Analyzing Compositional Data with R*. 2014: Berlin, Heidelberg : Springer Berlin Heidelberg.

3. van den Boogaart, K.G. and R. Tolosana-Delgado, *“compositions”: A unified R package to analyze compositional data.* Computers & Geosciences, 2008. **34**(4): p. 320-338.

4. Renaud, V., et al., *Comparison between open-site and below-canopy climatic conditions in Switzerland for different types of forests over 10 years (1998−2007).* Theoretical and Applied Climatology, 2011. **105**(1): p. 119-127.

5. Trigo, I.F., et al., *An assessment of remotely sensed land surface temperature.* Journal of Geophysical Research: Atmospheres, 2008. **113**(D17).

6. Trigo, I.F., et al., *Thermal land surface emissivity retrieved from SEVIRI/meteosat.* Ieee Transactions on Geoscience and Remote Sensing, 2008. **46**(2): p. 307-315.

7. Wan, Z., *New refinements and validation of the collection-6 MODIS land-surface temperature/emissivity product.* Remote Sensing of Environment, 2014. **140**: p. 36-45.

8. Freitas, S.C., et al., *Quantifying the Uncertainty of Land Surface Temperature Retrievals From SEVIRI/Meteosat.* Ieee Transactions on Geoscience and Remote Sensing, 2010. **48**(1): p. 523-534.

9. Bento, V.A., et al., *Improving Land Surface Temperature Retrievals over Mountainous Regions.* Remote Sensing, 2017. **9**(1): p. 38.

10. Zaitchik, B.F., et al., *Europe's 2003 heat wave: a satellite view of impacts and land–atmosphere feedbacks.* International Journal of Climatology, 2006. **26**(6): p. 743-769.

11. Baldocchi, D.D. and C.A. Vogel, *Energy and CO2 flux densities above and below a temperate broad-leaved forest and a boreal pine forest.* Tree Physiology, 1996. **16**(1-2): p. 5-16.

12. Carnicer, J., et al., *Contrasting trait syndromes in angiosperms and conifers are associated with different responses of tree growth to temperature on a large scale.* Frontiers in Plant Science, 2013. **4**: p. 19.

13. Otto, J., et al., *Forest summer albedo is sensitive to species and thinning: how should we account for this in Earth system models?* Biogeosciences, 2014. **11**(8): p. 2411-2427.

14. Duveiller, G., J. Hooker, and A. Cescatti, *The mark of vegetation change on Earth’s surface energy balance.* Nature Communications, 2018. **9**(1): p. 679.

15. Breuer, L., K. Eckhardt, and H.-G. Frede, *Plant parameter values for models in temperate climates.* Ecological Modelling, 2003. **169**(2): p. 237-293.

16. Li, Y., et al., *Local cooling and warming effects of forests based on satellite observations.* Nature Communications, 2015. **6**: p. 8.

17. Schleppi, P., A. Thimonier, and L. Walthert, *Estimating leaf area index of mature temperate forests using regressions on site and vegetation data.* Forest Ecology and Management, 2011. **261**(3): p. 601-610.

18. McGrath, M.J., et al., *Reconstructing European forest management from 1600 to 2010.* Biogeosciences, 2015. **12**(14): p. 4291-4316.

19. Poorter, L., et al., *Architecture of Iberian canopy tree species in relation to wood density, shade tolerance and climate.* Plant Ecology, 2012. **213**(5): p. 707-722.

20. Alkama, R. and A. Cescatti, *Biophysical climate impacts of recent changes in global forest cover.* Science, 2016. **351**(6273): p. 600-604.

21. Rebetez, M., et al., *Meteorological data series from Swiss long-term forest ecosystem research plots since 1997.* Annals of Forest Science, 2018. **75**(2): p. 41.
